# Recipe for Success: Suggestions and Recommendations for the Isolation and Characterisation of Bacteriocins

**DOI:** 10.1155/2021/9990635

**Published:** 2021-06-17

**Authors:** Ellen Twomey, Colin Hill, Des Field, Máire Begley

**Affiliations:** ^1^Department of Biological Sciences, Munster Technological University, Cork T12 P928, Ireland; ^2^School of Microbiology, University College Cork, Cork T12YT20, Ireland; ^3^APC Microbiome Ireland, University College Cork, Cork T12YT20, Ireland

## Abstract

Bacteriocins are bacterially produced antimicrobial peptides. Although only two peptides have been approved for use as natural preservatives foods, current research is focusing on expanding their application as potential therapeutics against clinical pathogens. Our laboratory group has been working on bacteriocins for over 25 years, and during that time, we have isolated bacteriocin-producing microorganisms from a variety of sources including human skin, human faeces, and various foods. These bacteriocins were purified and characterised, and their potential applications were examined. We have also identified bioengineered derivatives of the prototype lantibiotic nisin which possess more desirable properties than the wild-type, such as enhanced antimicrobial activity. In the current communication, we discuss the main methods that were employed to identify such peptides. Furthermore, we provide a step-by-step guide to carrying out these methods that include accompanying diagrams. We hope that our recommendations and advice will be of use to others in their search for, and subsequent analysis of, novel bacteriocins, and derivatives thereof.

## 1. Introduction

It is hypothesised that almost every microorganism produces at least one bacteriocin, including members of the archaea species [1]. Although many have been characterised, the full impact and role these bioactive peptides play in their ecological niches have yet to be elucidated [1–4]. It has been observed that at sublethal concentrations, bacteriocins facilitate communication between microorganisms occupying the same space, though when present in higher amounts, they exert bactericidal effects.

Bacteriocins are defined as bacterially produced, ribosomally synthesised peptides with antimicrobial properties against other bacteria. Alongside antibiotic production, the ability to make bioactive peptides is one of the oldest defensive measures microorganisms possess. Initially synthesised as inactive precursors, enzymatic cleavage at the N-terminal releases the active peptide. In some instances, the peptides undergo post-translational modifications prior to cleavage though this is not a universal trait [5, 6]. Although the classification of these peptides is under constant review [5, 6], the consensus is that these are small, bioactive peptides that are stable across a range of pH and temperature conditions. It has been most frequently observed that bacteriocins exhibit targeted activity [5, 6] against other closely related strains, typically those that reside in the same ecological niche. However, broad-spectrum bacteriocins with activity against a wider range of species have been identified, though to a lesser degree [7, 8].

Current trends in the market show that there has been an increase in the consumption of ready-to-eat (RTE) products, alongside a growing demand for minimally processed foods containing only natural additives [9, 10]. Keeping these aspects in mind, the food industry must still provide products with long shelf lives, high nutritional content, and a low risk of contaminants. The addition of bacteriocins to food products has been found to unify consumer demand with food safety needs.

Presently, only two bacteriocins have been released onto the market, both of which are applied as natural preservatives in food. These peptides provide a high standard of protection from microorganisms associated with foodborne illness and spoilage while also meeting the consumer demand for foods low in artificial preservatives. One such bacteriocin being used to achieve this is the *Lactococcus*-produced peptide, nisin. Nisin is the first bacteriocin to be generally regarded as safe (GRAS) and approved for use as a preservative in food [5, 11]. It is currently added to products sold in over seventy countries worldwide. The peptide has been found to successfully inhibit the growth of serious food pathogens including *Listeria monocytogenes* [12], which has been isolated from RTE foods, such as cooked meats and vegetables which are generally eaten as is (i.e., not heated or cooked further) [13–15]. Studies have also found that nisin improves the shelf life of a range of items, including dairy products and soft drinks by reducing the formation of compounds connected to spoilage while improving aroma and taste [16–18]. The second bacteriocin which has GRAS status and approved for market use is also derived from a lactic acid bacterium. This peptide, pediocin PA-1D, has shown similar activity against *L. monocytogenes* [19].

Given the potent activity that has been expressed against food contaminating organisms, research into the other potential applications of food-grade bacteriocins has been explored. In doing so, it has been found that these peptides possess bactericidal activity against a wide range of species, including clinically relevant organisms and drug-resistant strains such as methicillin-resistant *Staphylococcus aureus* (MRSA) [20–22]. Animal trials have confirmed that bacteriocins can retain their bioactivity and potency *in vivo*, indicating their potential as ingestible or injectable treatments [23–25]. The narrow spectrum of activity possessed by some bacteriocins (e.g., thuricin CD [26]), can be highly beneficial, as, unlike conventional antibiotics, the narrow spectrum bacteriocin can inhibit the target bacterium without collateral damage to the host's beneficial bacteria [5, 24, 27]. Investigations into the biomedical applications of bacteriocins expand far beyond their antibacterial properties. Current research indicates these peptides possess possible antiviral, anticancer, and spermicidal effects [28].

In this article, we aim to supply suggestions for the isolation and characterisation of bacteriocins based on the 25 years of experience and knowledge that our laboratory has accumulated, supplemented with relevant outside sources exploring the potential applications of bacteriocins. This will be done through the provision of step-by-step protocols and diagrams and will encompass the entire process from the collection of potential bacteriocin-producing strains from an environmental source to testing the potential applications of the purified peptide in complex media types. In doing so, we hope that our expertise can fill the gaps that conventional method reviews have overlooked to assist in the search for novel treatments that can drive back the tide of resistance and its impending threat to societal health and well-being.

## 2. The Isolation, Purification, and Characterisation of Bacteriocins

### 2.1. Selection of an Environmental Source of Bacteriocin-Producing Microorganisms

**Figure 1:**
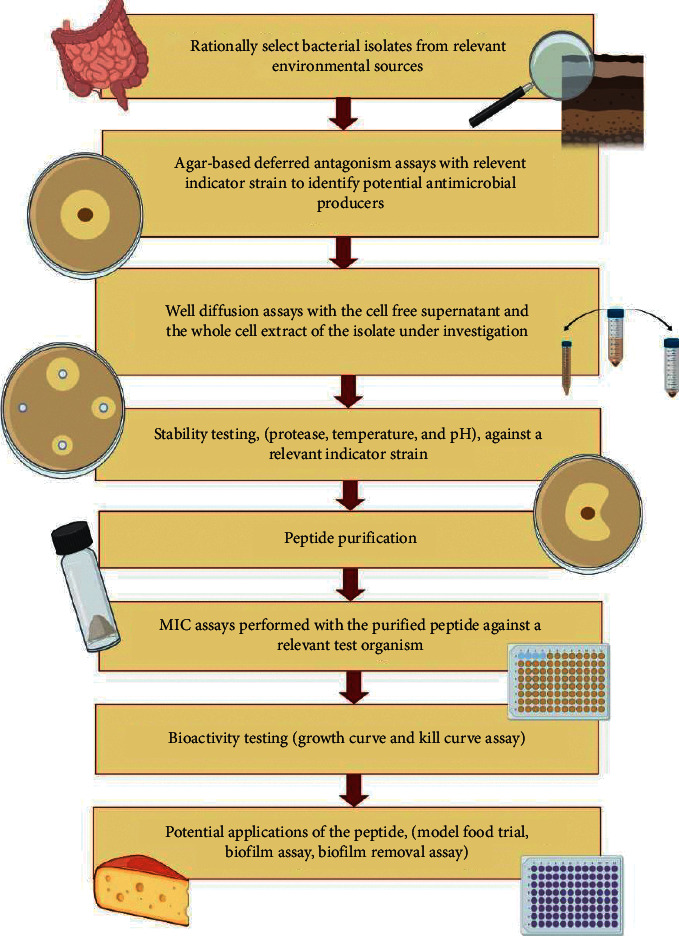
Overview of the approach employed in our laboratory for the isolation and characterisation of bacteriocins. This figure presents the protocols that will be discussed in the following article (e.g., temperature and pH stability studies, growth curves, and model food trials), created in BioRender.

As presented in Figure 1, the first step towards identifying a bacterium with antimicrobial potential is the rational selection of a location from which to isolate the microorganisms, and subsequent screening assays to identify strains with desirable activity. One of the most commonly used methods of identifying potential antimicrobial-producing strains is the agar-based deferred antagonism assay. Here, an overnight culture of an isolated strain under investigation is spotted onto a fresh agar plate and later overlaid and incubated with an indicator bacterium. Following incubation, the appearance of zones of clearing in the indicator strain growth in the overlaid agar is indicative of antimicrobial activity. Although deferred antagonism assays are the most commonly used method in our laboratory, a variety of other *in vitro* broth- and agar-based techniques can be employed when conducting a screening study. While broth-based assays, such as culturing of target strain in the presence of the isolate under investigation [29] or in the presence of its cell free supernatant [30], have been successfully applied to identify bacteria with bioactivity, agar-based methods are implemented more regularly. Commonly used agar-based techniques include such as spot-on-lawn assays, disc-diffusion, and well diffusion assays [31]. All three operate using similar principles, whereby the bioactive component diffuses through a solid medium and activity gauged by the inhibition of target strain growth. In the case of the spot-on-lawn assay, 10 *μ*L of cell free supernatant of the culture under investigation is spotted onto the surface of an agar plate which has been streaked evenly over the entire surface with a swab inoculated with the indicator strain. Disc-diffusion assays are performed with filter discs that have been soaked in the cell free supernatant of the culture under investigation. The saturated disc is then placed on the surface of an agar plate that has been streaked evenly over the entire surface with a swab inoculated with the indicator strain. The appearance of zones of clearing in the lawn of growth where the culture was spotted, or where the disc was placed, indicates bioactivity [31]. However, given that the peptide may not be produced in suitable quantities, these methods which use such small quantities of the potential antimicrobial may not be suitable for the initial screening process.

To prepare a bank of isolates for sampling, we recommend collecting strains from an environmental niche that has been chosen rationally. It is speculated that all strains of bacteria produce at least one bacteriocin [1], therefore isolating microorganisms with antimicrobial bioactivity from any habitat should, hypothetically, be a simple affair. However, it is important to consider the indicator strain the bacteriocin will be tested against when choosing an environmental source to collect strains.

A wide range of bacteriocins have been characterized, and although some broad-spectrum bioactive peptides exist, many possess a narrow spectrum of activity that is limited to closely related strains or other bacteria coexisting within the same habitat [24]. Taking into consideration the targeted activity spectrum some bacteriocins have, performing a screen using isolates obtained from an environment the target bacterium has previously been isolated from or is known to infect or contaminate, as this is likely to increase the chances of isolating a peptide with the desired bioactivity.

A study conducted by Rea et al. is a prime example of rational site selection of an environmental source when screening for bacteriocin-producing microorganisms [26]. *Clostridium difficile* is an opportunistic pathogen that typically invades the gut when the natural commensal microbiota has been damaged or disturbed. These disturbances can occur following a course of broad-spectrum antibiotics and result in chronic diarrhoea that can be particularly dangerous to the elderly and immunocompromised. Given the location of the infection site, Rea et al. postulated that the human intestines could house a bacterium that naturally inhibits the growth of this invading pathogen. For this reason, 30,000 isolates obtained from the faecal samples of adults were screened against a culture of *C. difficile*. To obtain a bacterium with targeted activity against *C. difficile* but that did not disturb the natural microbiota, Rea et al. aimed to isolate a spore-forming anaerobe from the bank [26]. The addition of an ethanol wash step and anaerobic diluent achieved this.

Of the 30,000 faecal-isolated strains, one isolate, later identified as a strain of *Bacillus thuringiensis*, was found to produce a novel, two-component peptide called thuricin CD which expressed potent activity against *C. difficile* [26]. Alongside its natural potency against the pathogen, the activity was highly targeted and left the remaining, natural microbiota of the gut unharmed. The large and diverse nature of the gut biome makes it an attractive target for the screening of bacteriocin-producing microorganisms and has led to many successful discoveries [7, 32]. For this reason, studies are currently underway in our laboratory that are investigating gut-derived isolates with the potential to inhibit clinically relevant pathogens, e.g., *L. monocytogenes* [33].

A second example showcasing the importance of rational sample site selection is seen in a study performed by O'Shea et al., which resulted in the discovery of a novel cationic bacteriocin [7]. The study aimed to identify an antimicrobial producer that could exert an inhibitory effect against gastrointestinal pathogens such as *Listeria* [34]. When designing a study to identify novel bacteriocin-producing microorganisms which target gut pathogens, O'Shea et al. opted to generate a bank of *Lactobacillus salivarius* strains for investigation. This *Lactobacillus* inhabits the same environment as *Listeria* and has previously been observed to exert immunomodulatory and anti-infective benefits to the hosts in whose gut they reside. Over the course of the study, ten strains, isolated from human and porcine intestinal samples, underwent genomic sequencing to identify possible bacteriocins hits. The analysis of *L. salivarius* DPC6502, originally isolated from porcine jejunal digesta, resulted in the discovery of a gene cluster encoding a novel antimicrobial. The purified peptide, called bactofencin A, showed potent activity against *S. aureus* and *L. monocytogenes*, comparable to that of lacticin 3147, another well-characterised bacteriocin previously studied in our laboratory [7].

Rationally selecting the environmental source of potential bacteriocin producers is a crucial step in designing a structured plan aimed toward the isolation of novel antimicrobial peptides, as is reflected in this study. By simply screening bacteria which colonise the same ecological niche the target strain is known to infect, a potentially novel treatment for a serious clinical pathogen is uncovered. However, O'Shea et al. also rationally designed their study by centring the project around a known bioactive species with demonstrable probiotic traits [7]. While the observation that these strains possess probiotic traits does not inherently mean a bacterium is a bacteriocin producer, the case of bactofencin A highlights why it may be worth investigating the cause behind the beneficial effect. In a similar vein to O'Shea et al., a study by O'Sullivan et al. performed genomic sequencing exclusively on a bank of coagulase-negative staphylococcal strains isolated from the skin [35]. This bank was created on the basis that many other coagulase-negative strains which make up the commensal microbiota, such as *Staphylococcus gallinarum* and *Staphylococcus hominis*, have been found to produce an array of antimicrobial peptides. The genomic analysis resulted in a set of 13 potentially novel bacteriocin-producing strains being identified [35], and one of which was found to be a naturally occurring variant of the well-characterised bacteriocin, nisin [36]. Nisin J showed activity against a range of microorganisms relevant to the medical and food industry, such as *S. aureus*, *Lactobacillus delbrueckii* subsp. *bulgaricus*, and *Cutibacterium acnes* [36].

### 2.2. Suitable Media Selection and Culture Conditions for Bacteriocin Production

Careful consideration of media type, media preparation, and culture conditions used in the screening process can greatly aid in the isolation of bacteriocins. If the media lacks suitable nutrients or culture conditions are unfavourable, the stain may not reach a suitable cell density [37]. The lower biomass would lead to the bacteriocin being produced in negligible quantities resulting in the failure to observe activity [37].

When collecting environmental samples, care must be taken in the choice of media and the culture conditions employed, as some bacteria may not survive the transition from their ecological niche to laboratory conditions. This could result in a reduction of cellular viability and strain diversity in the sample collected. To avoid bacterial loss, maximum recovery diluent can be employed to buffer and protect cells that may be susceptible to changing culture conditions [38]. Collins et al. successfully utilised this diluent when sampling from the intestinal contents, skin, and gills of fish [38]. Although the dilutions were plated on marine media 2216, the use of maximum recovery diluent gave additional protection to susceptible strains. As well as this, once the diluted samples were plated, Collins et al. incubated the samples for three days to ensure the recovered strains had a chance to proliferate.

Ensuring that a culture has a sufficient source of carbon has been proven to be an effective method of optimising peptide production [39–41]. Several studies attempting to ascertain factors that impact bacteriocin yields have confirmed the addition of glucose in concentrations ranging from 0.1% to 3.0% are effective at increasing the amount of bacteriocin in the culture medium [39–41]. Other sugars that have been studied for the same purpose include mannose, maltose, fructose, lactose, and sucrose, each with varying effects depending on the strain cultured [39, 40]. For some microorganisms, the addition of an organic nitrogen source is a necessity for peptide production, as the media alone may contain insufficient quantities, or the organism may be unable to derive biosynthetically from the other components in the media [42]. Zendo et al. discovered that when all nitrogen sources were removed from the culture broth, bacteriocin production from the strain *Enterococcus mundtii* QU 2 was completely nullified [43]. However, even the reduction of one nitrogen-containing element from the culture would result in a significant decline in observed activity [43]. Nitrogen sources that could be investigated for their ability to enhance bacteriocin production in a target strain include tryptone, peptone, beef extract, and corn steep liquor [40, 41, 43].

Finally, the addition of natural supplements that contain important macro- and micronutrients that might otherwise be missing from the culture, can also be tested to determine their impact on peptide production. Manzoor et al. found that the use of cheese whey (an industrial waste product and rich source of lactose, soluble proteins, lipids, and mineral salts) in the *Lactobacillus plantarum* culture medium leads to a significant increase in culture cell density, and in turn, bacteriocin production [40]. Cheese whey has been confirmed to enhance bacteriocin production in a range of other species including *Pediococcus*, *Bacillus,* and *Lactococcus* [44–46]. The addition of yeast extract to the culture medium has also previously been demonstrated to increase bacteriocin production in some species, including *Lactococcus* and *Bacillus* [47, 48]. Soy, molasses corn silage, skim milk broth, and corn steep liquor are further examples of natural supplements which have been analysed for their impacts on bacteriocin production [40, 41, 49, 50]. However, as with the tests concerning different carbon sources, the results vary depending on the strain selected [40, 41, 49, 50].

The composition of media and how it can be used to achieve suitable biomass and therefore substantial quantities of peptide should always be an important consideration. Appropriate carbon and nitrogen sources are applied in sufficient amounts during culturing for both bioactivity assessment assays and before purification. In the case of some bacteriocin-producing strains (*S. capitis* CIT060 [8], *S. capitis* APC 2923 [36], and *B. paralicheniformis* APC 1576 [38]), cell density and peptide production reached suitable levels without the need for media supplementation. However, *L. lactis* strains which produce nisin or lacticin 3147 [51–53] are grown in tryptone-yeast broth, and both of which are rich sources of nitrogen and important to the peptide production process in lactic acid bacteria [51, 52, 54].

When suitable media composition has been established, the ideal conditions under which to obtain high bacteriocin yields must be determined. Incubating the culture at a range of temperatures (above and below the temperature of the ecological niche it was isolated from) and adjusting the pH of the starting culture have both been found to affect the levels of peptide production [39–41, 55]. Another parameter to consider is the time point at which the most bacteriocin is produced. Well diffusion assays performed with the supernatant of cultures incubated to different extents have been used successfully to assess this factor [26, 55]. This assay not only prevents ending the incubation prematurely but can also indicates when the best time to stop culturing to prevent bacteriocin degradation if the culture conditions become unfavourable.

*L. lactis* is a lactic acid bacterium that is recognised for its production of bioactive peptides, such as nisin A and lacticin 3147. As is suggested by the name, this microorganism produces lactic acid through the fermentation of sugars in the media. At the late stages of the growth cycle, the build-up of lactic acid can reduce cell viability and create unfavourable conditions. To avoid such conditions which negatively impact the strain and the peptide, the incubation of *L. lactis* strains is stopped around the 16–20-hour mark and the buffer, *β*-glycerophosphate, is added to regulate pH [56]. It is important to consider that the ideal conditions for bacteriocin production may not be the ideal culture conditions. Therefore, contrarily, in the case of some strains, extending the incubation time to generate poor culture conditions can induce increased bacteriocin production. *Bacillus halodurans* C-125 is a spore-forming alkaliphile, originally isolated from soil [57]. It produces a two-peptide lantibiotic, haloduracin, which has previously been studied in our laboratory [57]. Investigations into haloduracin production by *B. halodurans* C-125 found that inducing sporulation caused a significant increase in the quantity of peptide produced [58]. This could be achieved by increasing the incubation period from 20 hours to 90 hours, which generated more unfavourable conditions in the culture [57, 58].

Finally, it has been observed that once removed from their habitat isolates may need to be stimulated to induce bacteriocin production. Moving away from the natural environment has previously been observed to cause a decline or inconsistencies in peptide production [59–61]. By inducing habitat-specific stresses through media composition and culture conditions, antimicrobial production can increase significantly [62]. Janek et al. documented this phenomenon when applying the stress of iron-limitation to a bank of 89 staphylococcal strains isolated from the nares [62]. Iron is an essential nutrient involved in many different bacterial processes [62, 63]. It can be taken up from the surrounding environment through bacterially produced molecules called siderophores, which are specifically designed for iron acquisition [62, 63]. Inducing iron limitation in the culture medium can increase bacteriocin production in a strain [62, 63]. By establishing iron-limiting conditions through the addition of an iron chelator, 2,2′-bipyridine to the media, Janek et al. observed that 52.5% of strains tested displayed an increase in antimicrobial activity against *M. luteus* and *S. aureus* [62].

This study also exposed the same strains to H_2_O_2_, which is produced by *streptococcal* competitors, and can induce bacterial cell death [62]. It was found that of the 84% of antimicrobial-producing strains within the bank, 18% displayed enhanced activity following exposure to 0.01% H_2_O_2_. Other environmental stress conditions that could be applied to isolate cultures include lowering the temperature to slow growth rate, oxygen saturation, or the addition of toxic compounds, like ethanol at a concentration of 1.0%, v/v, in the medium [64]. Each of these stresses (low temperature, oxygen saturation, and the addition of 1.0%, v/v ethanol) was found to increase the production of the bacteriocin, amylovorin L471, synthesised by *Lactobacillus amylovorus.*

The aforementioned conditions discussed relate to the production of bacteriocins for laboratory-scale analysis and therefore may not be suitable nor cost-effective for industrial or large-scale production. For example, pasteurized milk supplemented with yeast extract treated with protease is used as the substrate for the commercial production of nisin, as conventional media are unsuitable [65].

### 2.3. Suitable Indicator Selection

Indicator stain selection when screening for potential bacteriocins is critical, as the improper choice could result in the failure to observe antimicrobial activity. The target strain used as an indicator should preferably have pathogenic relevance, e.g., a strain of *L. monocytogenes* responsible for a serious outbreak of food-borne illness, strains of MRSA which pose a significant threat in healthcare settings, or the plant pathogen *Agrobacterium tumefaciens,* which causes financial loss through product damage [66]. This gives the activity of the bacteriocin relevance to market needs (see Table S1for suggested indicator strains and their corresponding growth conditions). Our experience in identifying and characterising bacteriocins has led us to find that the antimicrobial sensitivity of different strains within the same species can differ significantly, with some showing greater susceptibility than others. It is for this reason that we suggest that, when conducting the initial screening, multiple different strains of the same species be utilised as indicators to obtain the best overview of potential bacteriocin-producing strains within the bank of isolates.

The inclusion of numerous strains of the same target bacterium reduces the likelihood of an antimicrobial-producing bacterium being overlooked due to natural variations in antimicrobial susceptibility. An example of this was seen in a study by Lynch et al. when screening for bacteriocin-producing organisms within a bank of skin isolates. Six different *S. aureus* strains were employed in the screen (*S. aureus* NCDO1499, DPC5247, DPC5243, Newman, 5971, and RF122). One skin isolate in the bank that exhibited activity against four of the six *S. aureus* indicator strains was later shown to produce a novel bacteriocin. The inclusion of numerous strains of the same target organism reduces the likelihood of an antimicrobial producer being overlooked due to natural strain variations in antimicrobial susceptibility. Had the bacteriocin-producing isolate only been tested against one *S. aureus* strain, the antimicrobial activity of the strain may not have been uncovered.

Alongside the target indicator strain, we recommend including indicator strains with a high degree of antimicrobial sensitivity in the screen. Highly susceptible strains can assist in detecting antimicrobials that may be produced in low concentrations or indicate cultures with antimicrobial activity that are not effective against the main target pathogen of the study. This is a crucial step as it may reduce the likelihood of a novel producer in a bank going unnoticed. Examples of sensitive strains to employ in the screening process include *Lactococcus lactis* HP, *Lactobacillus delbrueckii* subsp. *bulgaricus* LMG 6901, *Corynebacterium fimi* NCTC 7547, and *Micrococcus luteus* [8, 67–71].

Even when activity against the target strain of the study has been observed, we suggest expanding the investigation of the isolate's activity by applying it against a wider, more diverse array of indicators. This could include autochthonous members of the human microbiome to ensure that they are not affected by the antimicrobial. In doing so, one can assess the spectrum of activity and obtain a more holistic overview of where the novel antimicrobial could be utilised. In doing so, one can assess the spectrum of activity and obtain a more holistic overview of where the novel antimicrobial could be utilised. Likewise, if the isolates under investigation do not show activity against the original, desired target indicator, they should not be disregarded. Instead, the potential activity of these strains should be explored further against a wider range of pathogen indicators to dictate if its activity has potential in other areas.

Actifensin is a defensin-like bacteriocin and the first of its kind observed to be produced by *Actinomyces ruminicola* [32]. The producing strain was isolated from a sample of sheep faeces [32]. Having confirmed the presence of antimicrobial activity against the target (*Lactobacillus delbrueckii* subsp. *bulgaricus* LMG 6901, an acid-resistant gut-dwelling bacterium), Sugrue et al. went on to assess the spectrum of activity of the purified peptide. Actifensin displayed broad-spectrum activity against Gram-positive microorganisms, including *C. difficile* and *L. monocytogenes*, widely known gut pathogens. The peptide also appeared effective against MRSA, indicating its potential to combat infections that occur outside of the habitat that the producing strain was originally isolated from. Studies such as this highlight the importance of expanding the field of indicators examined, as some bacteriocins could have hidden potential in areas outside of their ecological niche.

However, if looking to demonstrate that a particular niche is a prolific source of antimicrobial peptides, one must work in reverse: obtain the bank of isolates from the site under investigation, identify a relevant target pathogen, and then screen against it. The importance of identifying an appropriate indicator strain and applying it correctly is reflected in two studies with a similar objective, to identify novel antimicrobial-producing strains isolated from the human skin.

A study conducted by O'Sullivan et al. opted to target coagulase-negative staphylococcal strains in a large bank of skin isolates to identify those with potential activity against pathogens residing in the same niche, i.e., MRSA [35]. 90,000 isolates were obtained from seven superficial sites on the bodies of 20 individuals (140 sample sites total). These 90,000 colonies were isolated on either MSA or BHI agar plates. MSA in particular is a medium commonly used to isolate and identify staphylococcal species. Deferred antagonism assays were performed on the entirety of the bank against *Lactobacillus delbrueckii* subsp*. bulgaricus,* a Gram-positive gut-colonising microorganism, to identify strains with potential bioactivity. Following this screen, of the 90,000 isolates initially tested, 101 strains (0.12% of the bank) were shortlisted on the basis that they inhibited the growth of the target strain. Well diffusion assays (see Section 2.5for a description of this test) were then performed against the same target, using the neutralised supernatant of all 101 isolates to confirm that an antimicrobial substance was secreted into the surrounding media. This test further reduced the number of shortlisted bacteria to 25 (0.03% of initial sample size) based on the presence of a zone of inhibition. However, upon review, 12 of these shortlisted strains with inhibitory effects against the target were disregarded as they were deemed to produce zones that were too small. The remaining 13 strains, all found to be coagulase-negative staphylococci through 16S rRNA sequencing, were pursued for the remainder of the study. Genomic screening of the remaining isolates confirmed the presence of novel antimicrobial genes in all 13 strains [35].

While O'Sullivan et al. were successful in the attempt to identify novel bacteriocin-producing strains from isolates found on the skin, the improper use of indicator strains (using a strain associated with dairy as an indicator against skin-derived microorganisms) in the early screening stages potentially limited the number of novel producers discovered [35]. O'Sullivan et al. had an enormous starting bank of 90,000 skin isolates [35]. However, 99.98% of this was ruled out and discarded solely due to the inability of said strains to inhibit the growth of a single target. Given the typically narrow spectrum of activity bacteriocins possess, the selection of a target microorganism known to reside in an entirely different environment from the sampling site of the isolates investigated may cause an unfavourable bias in the study resulting in antimicrobial activity being missed. The antimicrobial effects against this single indicator were responsible for only 101 isolates being carried forward for further investigation and only 13 novel producers being isolated [35].

Comparatively, a study performed under similar conditions by Lynch et al. looked to evaluated antimicrobial production from 100 human-derived coagulase-negative staphylococcal isolates [8]. Although the starting bank was significantly smaller, Lynch et al. observed a higher percentage of isolates with bioactivity than O'Sullivan et al., following the performance of a deferred antagonism assay [8, 35]. This is primarily due to the range of indicator strains employed. Here, the deferred antagonism assays were performed against 24 indicators known to occupy similar ecological niches to the isolates tested, such as *M. luteus*, *Streptococcus*, and *S. aureus* which are members of the skin biome. 94% of isolates tested displayed antimicrobial activity against at least one of the indicator strains. One of these isolates, CIT060, was later found to produce capidermicin, a novel bacteriocin [8].

Two of the indicators used in this screen (*Geobacillus kaustophilus DSM7263* and *Geobacillus stearothermophilus* ATCC 12930) are environmentally derived bacteria that, similar to *L. delbrueckii* subsp*. bulgaricus,* would not be considered ideal target microorganisms for use in a screening of skin-dwelling bacteria [8]. CIT060, identified as a strain of *S. capitis*, did not inhibit the growth of *G. stearothermophilus* ATCC 12930. If this has been the only indicator Lynch et al. had opted to use in their screening, a potent bacteriocin producer would have been overlooked, which demonstrates the importance of indicator strain selection.

As mentioned earlier in this section, the susceptibility of a bacterium to an antimicrobial can vary between strains. For this reason, Lynch et al. used a variety of strains of the same species to ensure that an accurate overview of the antimicrobial spectrum could be ascertained [8]. This illustrates the second significant issue regarding indicator selection in the O'Sullivan et al. study. Isolates were shortlisted based on their ability to inhibit the growth of *L. delbrueckii* ssp. *bulgaricus* [35]. However, activity against only one strain (*L. delbrueckii* ssp*. bulgaricus* LMG 6901) was examined meaning that some isolates within the bank may have possessed activity against the species but were incorrectly disregarded simply because they did not show activity against this specific strain. We feel this is a critical step towards reducing the likelihood a novel producer is overlooked, one that could have been considered in the study by O'Sullivan et al.

### 2.4. Identification of Antimicrobial Activity through Agar-Based Deferred Antagonism Assays

A simple method commonly used to identify isolates with antimicrobial activity is the agar-based deferred antagonism assay (see Protocol S2(a), Figure S1, and Protocol S2(b)of Supplementary Material). As previously discussed in Section 2.1, this assay involves overlaying spotted isolate culture with 0.75% (w/v) agar inoculated with an appropriate indicator strain. The assay allows for the determination of antimicrobial action through the appearance of zones of inhibition in the indicator strain's growth.

As elaborated in Section 2.3, the appropriate selection of indicator strains is crucial. To best assess the number of antimicrobial producers in a bank, we recommend including a strain known to be sensitive to antimicrobials, such as *L. lactis* HP, in the screen to avoid failure to observe activity by strains that may produce antimicrobials in low quantities. We also recommend utilising multiple strains of the same species due to the variances in antimicrobial susceptibility can occur [8].

The observation of zones in the overlaid growth indicates the presence of antimicrobial action; however, they cannot be used to assess antimicrobial potency, as the size of the zone created can be misleading. As stated above, some strains may produce their antimicrobials in lower quantities than others, resulting in the zones produced appearing small. Zone size can also be reduced due to interfering components in the media such as charged sugar or sulphate residues binding to the antimicrobial, reducing its bioactivity [72–74].

The structure of the antimicrobial is another factor that can impact zone size as it determines how the peptide diffuses through a complex medium. This is a phenomenon previously documented by Healy et al. [75, 76]. In their study, Rouse et al. sought to investigate 12 bioengineered, mutated derivatives of the bacteriocin nisin, to determine if any had improved antimicrobial activity compared to the wildtype peptide [76]. Through agar-based deferred antagonism assays, Rouse et al. observed that all 12 derivative peptides showed enhanced antimicrobial action against at least one of the five indicator strains used; that is, they created larger zones of inhibition compared to those of the wildtype peptide. However, when the study transitioned to broth-based assays which removed the need for the peptides to diffuse through a complex medium, the enhanced potency of many of the derivative peptides disappeared. Instead, some of these peptides showed potency less than or equal to that of the wildtype. Rouse et al. surmised that the mutations (which all occurred at the hinge region of the nisin peptide) gave the bioengineered derivative peptides structures that permitted easier diffusion through complex polymers and thus showing artificially enhanced zones of inhibition during deferred antagonism assays [76].

### 2.5. Well Diffusion Assay

Well diffusion assays (WDAs) can be applied to assess the bioactivity of peptides and peptide-producing strains at different stages of the bacteriocin identification and characterisation process. For example, WDA can be undertaken prior to the purification process to best determine where most of the antimicrobial is concentrated, e.g., when the peptide is produced, is it excreted into the surrounding media or does it remain bound to the cell surface? By comparing the zones of activity obtained from the CFS and the whole cell extract (WCE) (solution containing the antimicrobial stripped from the cell surface), one can easily determine where the most peptide can be obtained from [8, 77] (see Protocol S3and Figure S2of Supplementary Material, for the preparation of the cell free supernatant and whole cell extract from an overnight culture). If conducting this test on the culture CFS, it is important to neutralise the pH of the supernatant prior to it being added to the wells. This will allow confirmation that any zones of clearing that are observed are caused by the presence of an antimicrobial compound and that they are not due to acid in the supernatant. WDA using the fractions obtained following HPLC can identify which of said fractions contain the active bacteriocin and which can be discarded. Also, WDA using the purified peptide can visualise its activity in complex media before performing broth-based MIC testing (as detailed in Section 2.4) [36, 75, 76].

Classifying where the bacteriocin is most concentrated is an important application of the WDA (see Protocol S4and Figure S3of Supplementary Material). Many purification process methodologies extract the peptide exclusively from the CFS, with no mention of the WCE component, e.g., the purification of epidermin, hominicin, and staphylococcin T [36, 49, 78–80]. In many instances, this may be suitable if the bacteriocin is solely present in the surrounding media [36, 49, 78–81]. However, it has been noted that bacteriocins, like those produced by lactic acid bacteria, can absorb to the producer cell surface [75, 77, 82, 83].

In a study to investigate methods to obtain improved bacteriocin from cultures, Yang et al. found that a low pH would cause bacteriocins to be released from the surface of cells into the surrounding media [83]. By incubating the cells at a low pH (pH 2.5) for one hour with agitation, a higher yield of the bacteriocin would be obtained than from extracting from the cell free supernatant alone [83]. Several bacteriocins, including lactocin AL705 and pediocin F, have been purified by this method [82, 83]. Therefore, to get the most out of the purification process, one will need to combine peptide extracted from the CFS and WCE of the overnight culture. If this is neglected, the culture will give low yields, resulting in the need for multiple purifications to collect suitable quantities of pure bacteriocin [8, 26, 77].

Our laboratory uses a similar principle when extracting antimicrobial peptides from *L. lactis*, the bacterium which produces nisin and that has been bioengineered to produce enhanced bioactive nisin derivative peptides. Although much of the nisin peptide is present in the culture medium, a significant portion remains bound to the cell surface, requiring it to be stripped away and combined with the peptide in the CFS before purification [75, 77]. We have achieved this by incubating the cell pellet of an overnight culture in a solution of 70% isopropyl alcohol, 0.1% trifluoracetic acid, and distilled water. Trifluoracetic acid is a key component. A strong acid, it generates a suitable pH (pH ≤ 2.0) and goes on to form a complex with peptides, assisting in peptide detachment from the cell surface [84, 85].

### 2.6. Assessment of Antimicrobial Activity through Agarose-Based Radial Diffusion Assays

As stated in Section 2.4, the sizes of clearings in the overlaid indicator strain growth can be reduced by interfering components in the media, e.g., charged sugar or sulphate residues [72–74]. These interfering agents can interact with the antimicrobial and inhibit its activity. To overcome these unwanted interactions, it has been observed that performing agarose-based radial diffusion assays (see Protocol S5and Figure S4of the Supplementary Material) with the CFS of the isolated strain to be a suitable solution [51].

Agarose reduces unfavourable electrostatic interactions with the bacteriocin and the media, and the nutrient-limiting conditions of the seeded base layer reduce the ability of the indicator bacterium to reproduce [72–74]. A second crucial reason for performing agarose-based radial diffusion assays is the visualisation of antimicrobial activity against Gram-negative indicators.

The outer membrane that Gram-negative microorganisms possess acts as a bulwark, inhibiting the activity of some bacteriocins by blocking their access to the cell wall [51]. Agarose-based radial diffusions are incredibly sensitive which allows them to be used to visualise weak or small zones of clearing in the growth of the indicator strain. This method has successfully been employed to detect activity against Gram-negative strains which were potentially sensitive to a bacteriocin (nisin) which has previously only shown activity against Gram-positive strains. Experimental parameters can be adjusted to render Gram-negative cells susceptible to bacteriocins that typically only affect Gram-positive microorganisms (sublethal thermal exposure, the addition of chelating agents, and osmotic shock [51]). However, agarose-based radial diffusion assays are capable of visualising the antimicrobial interactions between bacteriocins and Gram-negative bacteria without the need to induce susceptibility, even if the degree of growth inhibition by the peptide is low. During an agarose-based radial diffusion assay, the test bacterium is exposed to only the bacteriocin and a buffer before the overlay occurs, which slows indicator strain growth and reveals sensitivity to the antimicrobial [51].

This has previously been demonstrated by Field et al. when screening nisin derivative peptides for enhanced activity [51]. Agarose-based radial diffusion assay revealed that nisin, along with several novel nisin mutants showed activity against Gram-negative pathogens *Cronobacter sakazakii, E. coli*, and *Salmonella*, without the addition of any other compounds that would induce susceptibility. Combined, we feel the addition of the results from agarose-based radial diffusion assays to those obtained from the agar-based diffusion assays and allow for improved assessment of antimicrobial producers in a bank of isolates.

### 2.7. Purification of Peptides

Before beginning purification, we recommend performing WDAs on both the CFS and WCE of the strain under investigation, to assess when the highest concentration of peptide resides, as previously described in Section 2.5. Extracting antimicrobial peptide from both the CFS and WCE components [77] will assist obtaining optimum yields during the purification process (see Protocol S6of Supplementary Material).

When setting up the overnight culture for purification, the culture medium and incubation parameters must be optimised for bacteriocin production. When performing purifications on nisin and nisin derivative cultures 2 L of broth inoculated with 1% overnight culture was found to produce suitable quantities of peptide [36, 77]. It is important to consider that this volume may not be adequate if the strain produces the peptide in low concentrations. In some situations, it may also be more than is required. Previous studies have performed purification on smaller volumes (1 L overnight culture [26]), and other studies have required larger amounts (3 L volumes [8]). Ensure that the broth has been supplemented with all the additional nutritional requirements (e.g., carbon source, nitrogen source, and micronutrients) and buffers it requires. Prior to culturing the bacteriocin-producing strains and HPLC, pass the broth through a column of Amberlite XAD-16N beads (Sigma-Aldrich) [8, 26, 36]. This clarifies the media by removing hydrophobic and charged components that could form nonspecific interactions with the peptide [73] and interfere with downstream purification processes.

Having harvested the bacteriocin from both the CFS and WCE, the two elements can be pooled to continue the purification process. Our laboratory purifies bacteriocins through HPLC using a Phenomenex C12 Reverse-Phase (RP) HPLC column (Jupiter 4*μ* Proteo 90 Å, 250 × 10.0 mm, 4 *μ*m) [8, 75, 77]. To facilitate the purification of nisin and its mutated derivatives, we employ a gradient of 30–50% acetonitrile and 0.1% trifluoroacetic acid. This however will not be suitable for all bacteriocins, particularly if it is the first purification of a novel peptide and the percentage of solvent required for elution is not known. For the initial purification, a gradient of 5%–80% organic solvent and acid should be employed to guarantee that everything has been eluted from the column. Upon completion of the initial HPLC process, WDAs should be performed using each fraction from the run to determine which contain the active peptide. Correlating the results of the well diffusion assay and the peaks graphed during the HPLC process, the percentage of solvent required to elute the peptide can be calculated. Future purifications can be tailored once the percentage solvent is known, i.e., no longer have to start at such a low concentration (5%) and run until such a high concentration (80%) if it is known that the peptide elutes around 30–40% solvent.

Having determined the retention time of the peptide, the relevant fractions can be combined and subjected to further rotary evaporation, as any remaining organic solvent present must be removed [75, 77]. The remaining liquid can then be lyophilised to preserve the integrity and to suitably store the peptide [75, 77].

The purification method discussed has been employed in a number of experiments in our laboratory to isolate bacteriocins from several species (*Lactococcus*, *Staphylococcus*, and *Bacillus*) [8, 36, 38, 52, 69]. It may not, however, be suitable for all peptides. For this reason, if the aforementioned method is unsuccessful, alternate options such as chemical solvents, ion exchange, and gel chromatography [86] could be explored. Ammonium sulphate has previously been used as an effective chemical solvent to precipitate peptides from bacterial cultures without destroying bioactivity [87]. Another method used for purification of peptides from large volumes of culture is ion exchange [88]. The application of the bacterial culture to the appropriate column results in the rapid purification of peptides with a high degree of purity.

A combination of RP-HPLC and MALDI-TOF mass spectrometry can be used to determine if a purified substance obtained from the pooled active fractions contains a single, active bacteriocin, or if multiple peptides are present. Following purification, HPLC chromatograms of the purified sample should be examined for the presence of multiple peaks, indicative of more than one peptide has been obtained [89]. Moreover, purity of the sample can be determined by mass spectrometric analysis. Similar to HPLC analysis, a pure sample should reveal the presence of a single, clear peak. If it has been identified that multiple peptides are present, individual peptides can be separated by optimising HPLC conditions. This can include the manipulation of solvent type and temperature to assist in achieving separation of peptides which coelute [90]. Tricine-SDS-PAGE analysis in 16% acrylamide gels and 10% acrylamide gels with visualisation by silver staining is another method by which purity of a bacteriocin fraction may be assessed [91].

In conjunction with peptide purification, genomic analysis for the identification of biosynthetic gene clusters involved with bacteriocin production can be performed to determine if the peptide isolated is novel and can assist in determining the structure. Genome mining tools such as BAGEL, BACTIBASE, and Anti-SMASH can be applied to identify genes of interest, such as genes associated with immunity, regulation, transport, and peptide modifications [92–95]. BLAST searches and alignment tools such as ClustalW can also aid in determining if the peptide isolated is novel or not. Direct matches to gene clusters of previously characterised peptides may indicate that the purified peptide has been identified in the past. However, if the genes of strain under investigation only show similarity to bacteriocin associated genes, then it is possible that a novel antimicrobial has been found.

### 2.8. Bacteriocin Susceptibility and Stability Assays

Several traits including thermostability, stability over a range of pH conditions, and protease enzyme sensitivity have been attributed to antimicrobial peptides [96, 97]. It is important to examine if the antimicrobial being investigated possesses stability under a range of stressful conditions to ascertain both its stability spectrum and to propose, based on its sensitivities, which products or environments it could be best applied. Bacteriocins are proteinaceous and are therefore sensitive to protein degrading enzymes [96, 97]. Exposure to proteinases can either reduce or completely inhibit their bioactivity [96, 97]. Protease susceptibility assays have been successfully employed to assist in the characterisation of novel bacteriocins in numerous studies [8, 26, 36, 38] (see Protocol S7(a)and Figure S5or Protocol S7(b)and Figure S6of Supplementary Material).

Incubating the CFS, purified peptide, or partial/crude peptide extract with a protein-degrading enzyme could indicate if the antimicrobial activity previously observed in agar and broth-based assays is due to the presence of a bacteriocin or a conventional antibiotic. Antibiotics are secondary metabolites with chemical structures, ergo, and they are unaffected by enzymes with proteolytic activity. Following incubation, if bioactivity of the protease exposed sample is reduced or inhibited, it can be concluded that the active agent was proteinaceous in nature. The secondary purpose of this test is to build a complete profile of the peptide and its nature regarding durability and susceptibility to proteases. To ensure that the entire spectrum of sensitivity is gauged, a variety of enzymes, including proteinase K, *α*-chymotrypsin, and pepsin, could be used against the peptide [8]. Given their potential application as natural preservatives in food, protease susceptibility profiles of the peptide should be established as it is reasonable to assume that the bacteriocin under investigation could come into contact with digestive enzymes or other protein degrading enzymes that are present in some food products.

It is critical that when conducting bacteriocin pH and temperature stability tests, suitable ranges of both experimental parameters are examined given the range of environmental conditions bacteriocins could face when applied to foodstuffs or as medicines (see Protocol S7(c)and Protocol S7(d)of Supplementary Material to undertake pH stability and temperature stability testing, respectively). The thermostability of potential bacteriocins can be ascertained by incubating the CFS or purified peptide at temperatures from as low as 0°C to 121°C, for 15 minute periods [26, 35, 38, 52]. Well diffusion assays on the heat-treated peptides against the target strain can then be performed to determine if the bioactivity has been affected. These temperatures reflect food storage, sterilisation, and cooking temperatures that could denature and inactivate the peptide. Likewise, this encompasses the range of temperatures a pharmaceutical drug product may be exposed to during storage and sterilisation. The stability range of the peptide could be insightful as when to add or apply a bacteriocin during the food preparation or drug manufacturing process. If the bacteriocin is stable under conventional autoclave conditions (121°C, at 15 psi for 15 minutes with moist heat), it would simplify the addition of the peptide to the production process, as it could be included in the terminal sterilisation process.

The CFS containing the bacteriocin or the resuspended purified peptide should be adjusted in a pH range from pH 2 to pH 11 [26, 35]. This too will build a complete picture of bacteriocin stability under different physiological pHs. Not all foodstuffs have a neutral pH, e.g., fermented foods or soft drinks. If the bacteriocin retains its activity or becomes denatured, it would be indicative of which environments the peptide may or may not be suited to. Well diffusion assays using the pH-adjusted samples against a relevant target strain can be used to characterise the effects these altered conditions have on peptide bioactivity. As the first bacteriocin approved for consumption, nisin has been well studied and characterised. Most stable at a low pH, the peptide's solubility has been found to decrease as pH increases [98, 99]. Despite high thermostability during the sterilisation and preparation process, high storage temperatures over an extended period of time (25°C–30°C for eight weeks) greatly reduced peptide viability [99]. Understanding the stability and durability of a bacteriocin can help assist in understanding its application and how it can be used to improve a product or patient needs.

It should be considered though that this sensitivity to environmental conditions is one of the primary issues facing the *in vivo* activity of bacteriocins. Despite showing stability when tested using broth and agar, a bacteriocin may act differently on or in the body. In animal trials, it has been observed that when consumed orally, lacticin 3147 did not survive transit through the gut [100]. Exposure to proteases and unfavourable pH conditions inactivated the peptide before reaching the target site. On the other hand, when used by the intravenous and interperitoneal route, lacticin 3147 retained activity [101]. Although a thorough assessment of protease and pH susceptibly can indicate peptide suitability under laboratory conditions, further studies are necessary to address suitability for *in vivo* use.

### 2.9. Minimal Inhibitory Concentration (MIC)

A method routinely used to determine the MIC of an antimicrobial is that which is described by both the European Committee on Antimicrobial Susceptibility Testing (EUCAST) and the Clinical and Laboratory Standards Institute (CLSI) [8, 12, 102–104] (see Protocol S8and Figure S7of Supplementary Material). An MIC, as defined by EUCAST, is “the lowest concentration of the agent that completely inhibits visible growth as judged by the naked eye, disregarding a single colony or a thin haze within the area of the inoculated spot” [104]. Therefore, visual inspection of plates following incubation is the only required method to determine the MIC.

If conducting the MIC assay in a plastic 96-well microtiter plate, it is best practice to incubate a sterile solution of 1% (wt/vol) bovine serum albumin (BSA) dissolved in phosphate buffer in each of the wells, for 30 minutes at 37°C [51, 77, 105]. This prevents nonspecific protein absorption to the plastic surface, which could reduce the bioactivity of the peptide. As previously mentioned in Section 2.3(Suitable Indicator Selection), we recommend careful selection of the target strain for this assay. Attempting to determine the activity of the peptide against a bacterium outside of its spectrum of activity (as previously confirmed in agar-based diffusion assays) may allude to inactivity, when in reality, the peptide is simply being misapplied. It is important to note that some peptides may have enhanced diffusion through complex media compared to others. The shift from solid, complex media to broth, which eliminates the need for diffusion, will reveal which peptides possess potent activity against the target indicator strains and which are superficially enhanced [75, 76]. This enhanced diffusion can result in a huge disparity between the results observed in agar-based tests and those performed in broth, as described in depth in Section 2.4.

MIC assays are end-point tests, meaning that a singular result, or set of results, is obtained once the reaction (the incubation of the peptide with the target strain) has been stopped. At the end of the incubation period, the potency of two peptides may appear to be similar (twofold difference in MIC value) or equivalent. However, this end-point result may not accurately reflect the activity throughout the incubation, and we therefore suggest performing “kinetic tests,” such as growth and kill curves. These kinetic assays can be used to monitor the bacterial cell death and recovery rate over the extent of the target strain's exposure to the peptide. This is discussed in greater detail in Section 2.10(Growth and Kill Curve Assays). When performing MIC assays, it is important that multiple strains of the same species be investigated given that antimicrobial susceptibility can vary between strains [8, 56]. This can lead to an improper judgement on the antimicrobial spectrum of the peptide [8, 56].

Although the MIC assay is the most widely utilised gold standard model, it requires purified bacteriocin preparations and activity is expressed as mg/ml. The activity units (AU) assay is analternative method that can be used to express activity of CFSand activity is presented as activity units per millilitre (AU/mL) [35]. One bacteriocin unit is defined by Zhang et al. as “the reciprocal of the highest dilution that showed a clear inhibition zone” [106].

### 2.10. Growth and Kill Curve Assays

As an end-point assay, MIC tests cannot give insight into the viability of cells throughout the exposure to the antimicrobial, as only one result is taken at the end of the incubation period. It is for this reason that growth curve assays (see Protocol S9and Figure S8of Supplementary Material) and kill curve assays (see Protocol S10and Figure S9of Supplementary Material) should be performed. These assays can visualise the effects the peptide has on lag time and bacterial cell recovery.

A study conducted by Field et al. sought to compare the antimicrobial action of a novel bioengineered nisin derivative to that of the wildtype peptide [107]. A kinetic kill curve assay was performed using the bacteriocin at lethal levels against the test microorganism, where the kill effects of the wildtype peptide were compared to that of the derivative at three different time points of the incubation period (20 minutes, 40 minutes, and 60 minutes) [107]. Plate counts performed using samples of the culture taken at these different time points revealed how the derivative peptide both caused a greater reduction in test bacterium cell count, while also suppressing cell recovery for a greater period of time compared to the wildtype [107]. This example showcases how kinetic assays can provide a more in-depth perspective into the antimicrobial effects of a bacteriocin that end-point assays cannot.

When conducting a kill curve assay, it is best practice to monitor several time points to gain a complete overview of bacteriocin kill of target cells. Serially diluting and plating samples at regular intervals and comparing colony counts to samples of the test bacterium cultured in the presence or the absence of the peptide will permit the plotting of a graph that captures the differences in lag phases. Therefore, it is important to incorporate these tests into the analysis of peptide activity. In growth curve testing, effects of the bacteriocin at sublethal levels on test bacterium lag, recovery, and growth can be observed over 24 hours using an automated plate reader [77].

### 2.11. Model Food Trials

To this point, the discussion has focused on examination of the antimicrobial activity of bacteriocins in conventional laboratory media. However, although a bacteriocin may show potent activity against target strains under these conditions, it has been noted that when applied to a more complex environment, bioactivity can be reduced or completely negated. Model food trials can be carried out in order to examine the bacteriocin's antimicrobial activity in various food types.

When conducting a model food trial, it is crucial that a relevant target strain is selected to ensure the trial that reflects real-life conditions as closely as possible (see Protocol S11(a)and Protocol S11(b)of Supplementary Material). If a specific target has not already been chosen, then the following should be taken into consideration. To attain market relevance and mirror real world issues food production companies face, select a bacterium that has previously been found to contaminate food, e.g., *L. monocytogenes* L. [108], *E. coli* [109], or *C. sakazakii* [110]. Although a surrogate strain can be used in place of a pathogenic test microorganism, we recommend using a pathogen, specifically one that has been isolated from the source of an outbreak, e.g. *L. monocytogenes* isolated from a listeriosis outbreak or *Salmonella* strains linked with salmonellosis outbreaks. Bacteria have also been known to enter the food chain through contaminated processing food machinery or unclean food processing environments [111]. Sampling from these environments would also provide suitable antimicrobial targets. Finally, as mentioned in previous sections, the degree of antimicrobial susceptibility can vary between strains of the same species. This phenomenon has as previously demonstrated by Begley et al. who observed the stain variable nature of nisin tolerance in a collection of *L. monocytogenes* strains [112]. To obtain a more accurate overview of peptide activity and avoid the pitfalls of testing against a highly sensitive or highly resistant strain, we recommend testing it against a range of strains from the same species.

Having identified a suitable pathogenic target strain, a medium that the pathogen is known to contaminate must be selected for the trial to ensure that the conditions provided are both relevant to the market and as accurate to real-life contamination conditions as possible; for example, pathogenic *L. monocytogenes* is known to contaminate chocolate milk [108], *E. coli* has previously been found to contaminate apple juice [109], and *C. sakazakii* is known to cause infant formula contamination [110]. When inoculating the food sample with the indicator strain for the trial, the concentration of CFU/mL must be considered. The typical concentration of pathogenic cells in a food sample may be too low to detect through plating or may not be a suitable quantity to show the effects of bacteriocin exposure. For example, the natural concentration of *L. monocytogenes* contamination in food is typically less than 10^2^ CFU/mL [113]. To facilitate detection, we suggest inoculating at a higher concentration, as close to the real-life inoculum as possible, but with a quantity that would still give a viable CFU/mL result when plated. Previous model food trial studies have used inoculums, standardised to a final concentration of 10^5^ CFU/mL, in several food models [76, 114], but the concentration required may vary depending on the strain used.

Food trials have been conducted to test the capability of nisin and its bioengineered derivatives to inhibit the growth of relevant pathogens in a range of foodstuffs, e.g., chocolate milk, the gelling agent carrageenan, chicken noodle soup, frankfurter meat, infant formula, and apple juice [12, 76, 105, 114]. As previously stated, while a peptide may display promising antimicrobial action under laboratory conditions, the complex environment it may be applied to could contain inhibitory factors that reduce or completely negate bioactivity. Components such as complex carbohydrates, lipids, proteins, and the pH of the environment can interfere with and reduce peptide functionality [76, 115].

One particular study conducted by Campion et al. highlighted how bacteriocin activity can differ depending on the food to which it is added [114]. The study in question assessed the bioactivity of nisin and two nisin derivative peptides in several foodstuffs, contaminated with a relevant pathogen. In broth-based assays, the wildtype nisin A peptide had a MIC of 3.75 *μ*M (12.57 mg/L) against the target strain, *C. sakazakii*. The wildtype was then incubated with *C. sakazakii* in spiked infant formula at a concentration of 60 *μ*M (>100 mg/L). Despite the high concentration of peptide, no reduction in *Cronobacter* CFU/mL was observed. In this instance, while the peptide was found to be effective against the pathogen in broth, the activity appeared to be almost completely inhibited by the complex medium. In broth-based kill curves, the derivative peptides (nisin V and nisin S29A) were significantly enhanced compared to the wildtype [114]. Enhanced activity was most notable when the peptides were used in conjunction with food-grade oils, e.g., carvacrol. However, when applied to the infant formula, the derivative peptides demonstrated activity equivalent to that of the wildtype, despite the addition of carvacrol oil [114].

The reduction or inhibition of bioactivity that can occur when applied to a model food system, as observed in this study, showcases the significance of these tests in the peptide characterisation process. Campion et al. noted that although inactive when tested in spiked infant formula, the activity of the nisin A peptide was retained when cultured in commercially prepared apple juice, causing a log reduction in the indicator, *E. coli* O157 [114]. We recommend that multiple food types of differing compositions known to be contaminated by the target strain be investigated, as although the peptide may be inhibited in one product, activity may be substantially improved in another. As stated previously, antimicrobial susceptibility among strains within the same species can vary [8, 56]; therefore, we highlight the need to incorporate multiple strains into these studies to accurately assess the spectrum of activity of the peptide in a complex medium.

### 2.12. Biofilm Assays

Biofilms are bacterially produced exopolysaccharide matrices that permit planktonic cells to adhere to biotic and abiotic surfaces [116]. These mucous layers prevent the cells from being removed and protect them from harmful external factors such as chemicals, e.g., disinfectants [117, 118], and antibiotics [119]. The ability to form biofilms allows organisms to create persistent sources of contamination that affect many different industrial sectors. Companies involved in the preparation and packaging of food face the issue of persistent biofilm formation. Microbial biofilms have been observed to form on the surfaces of steel pipes [120], on equipment surfaces (e.g., tanks [121]), on submerged surfaces [122], and within packed food itself [123]. Previously bound cells can be released from the matrix [124, 125], allowing microorganisms to pass along pipelines or spread across surfaces, contaminating products poststerilisation [120, 121]. These newly planktonic cells can potentially reach foods that are distributed, sold, and consumed. With the demographic of aging and immunocompromised people in society increasing, the threat presented by contaminated food grows year by year [9].

In hospital settings, biofilms are equally demanding of attention. Given the opportunity to invade the body, e.g., through the implantation of contaminated medical device materials, bacteria can form a biofilm within the host, creating a persevering source of infection [119, 126]. These infections are very difficult to treat, generating increased risk and suffering for patients. The development of biofilms has also been observed in the water supply and pipe networks of pharmaceutical manufacturing companies [127], which is particularly worrisome given the need for complete sterility in the manufacturing and packing processes for such products. If a bacteriocin has been found to inhibit the growth of clinically relevant pathogens associated with biofilm formation, exploring the peptide's ability to reduce or prevent biofilm development on different surfaces could lead to a better overview of its potential application.

The stationary microtiter plate method can be used to assess the biofilm growth of target strains [56, 77, 128, 129] (see Protocol S12and Figure S10of the Supplementary Material). This assay has successfully demonstrated that nisin A and its bioengineered derivatives can reduce the formation of biofilm both on plastic and medical device material surfaces [56, 77, 128, 129].

When selecting strains for biofilm inhibition studies, it is important to take into account where a strain has been isolated from, e.g., cells persistently found contaminating food, strains isolated from tanks, pipes, or drains in the food or pharmaceutical industries, or taken from the surface of an explanted medical device. The biofilm-forming capabilities of each strain should then be investigated prior to bacteriocin exposure, which can be done by studying the biofilm coverage of a microtiter plate (round well, flat-bottom plates with a lid to ensure sterility), staining with crystal violet, and measuring the absorbance of each well. Our laboratory has previously allowed strains to cultivate over a 48-hour incubation period when assessing biofilm production [77, 128]. However, maximum biofilm growth may be achieved much earlier than this, depending on the strain under investigation. A factor that can interfere with the assessment of biofilm formation is the spontaneous erosion and degradation of the exopolysaccharide matrix. It is believed factors such as shearing forces, the age of the biofilm, and declining culture conditions can result in this breakdown [124, 125, 130, 131]. Therefore, to avoid incorrectly classifying a bacterium as a weak or moderate biofilm former, we suggest inspecting biofilm formation over a range of time points (20 hours–48 hours), to ascertain when the most biofilm has been generated. Biofilm-forming strains can be classed as “weak,” “moderate,” or “strong,” according to the criteria established by Christensen et al. [132]. We advise opting to use a “strong” biofilm-forming strain to best gauge the proficiency of the bacteriocin.

A recent study by Twomey et al. demonstrated the potential of bacteriocins to inhibit the formation of biofilm on medical device substrates, hinting at the possible biomedical and pharmaceutical applications of antimicrobial peptides [56]. This study aimed to identify bioengineered nisin derivatives with enhanced bioactivity compared to the wildtype nisin A against strains of *S. epidermidis*, previously isolated from the blood of patients in a hospital setting. Twomey et al. observed that nisin A and its bioengineered derivative, M17Q, not only reduced the formation of two strong *S. epidermidis* biofilm-forming strains on plastic microtiter plate surfaces, but also on the surfaces of three catheters materials (rubber, polyvinyl chloride, and polyvinyl resin), as well as stainless steel, a material which composes some indwelling prosthetic devices [56].

When culturing target strains, it is recommended that the broth be supplemented with 1% (w/v) D-(+) glucose, as the addition of glucose has previously been found to enhance biofilm formation [56, 133]. Once biofilm has formed and the plates are being examined for growth, caution should be exercised when rinsing or transferring liquids from the wells. Scraping the wells with a pipette tip or using unnecessary force can result in the unintentional removal of bound material which will impact the absorbance reading obtained and the overall assessment of biofilm formation or inhibition. When incorporating the antimicrobial peptide, we recommend doing so at a range of concentrations, e.g., the MIC, 2 × MIC, and 1/2 MIC [56, 134]. This will allow for the assessment of bioactivity both above and below the MIC and can indicate a suitable concentration that may inhibit biofilm formation completely. Lynch et al. recently employed this method to assess the anti-biofilm-forming capabilities of the bacteriocin capidermicin against a strong biofilm-forming strain of *L. monocytogenes* (*L. monocytogenes* EGDe) [134]. At concentrations 2 × MIC, 1 × MIC, and 1/2 × MIC (1 × MIC = 3.75 *μ*M), capidermicin was capable of causing a significant reduction in biofilm formation compared to the untreated control.

Antimicrobials should be investigated not only for their ability to inhibit biofilm formation but to remove pre-established biofilm, given the nature of such infections (see Protocol S13of Supplementary Material). To assess the capability of a peptide to remove preformed biofilm, the biofilm-forming strain must be allowed culture in the absence of the antimicrobial until maximum biofilm growth has been obtained. Following incubation, carefully remove the culture and replace with peptide resuspended in fresh, relevant broth. Incubate the peptide-exposed biofilm for 18–24 hours. In the same context as the biofilm inhibition test, we recommend that the peptide be tested at a range of concentrations. Given the difficulty to remove preformed matrices, it is likely that large concentrations of the peptide may need to be used. Lynch et al. also tested the ability of the bacteriocin capidermicin to remove pre-established *Listeria* biofilms on a plastic microtiter plate surface [134]. Using a range of concentrations from 1 × MIC to 8 × MIC (1 × MIC = 3.75 *μ*M), Lynch et al. noted a significant decrease in bound biofilm compared to the untreated control in five strong biofilm-forming strains (*L. monocytogenes* CD749, Ts45, F2365, EGDe, and OB001102), following 24 hours of incubation with the bacteriocin. The same caution should be exercised when transferring liquids and changing cultures as when analysing biofilm formation, as scraping away biofilm through careless pipetting can lead to the improper assertion that a peptide may be capable of removing preformed biofilm.

As stated above, the removal of pre-established biofilm is a different and significantly more difficult task than biofilm inhibition, given the strongly adherent properties of the matrix. However, it has been found that bacteriocin peptides are capable of penetrating the slime layer. Although they cannot remove the biofilm completely, the peptides are still able to exert an antimicrobial effect on the cells within and reduce the threat of infection or contamination.

The rapid colorimetric XTT assays (performed with the chemical 2,3-bis[2-methyloxy4nitro-5-sulfophenyl]-2H-tetrazolium-5-carboxanilide) can be used to determine the viability of cells within the biofilm [77]. The colour change detected through this assay is a result of the tetrazolium salt XTT being reduced, a conversion that only occurs in viable cells. The absorbance observed directly correlates to the number of live cells present. Field et al. have utilised this test to determine if exposure to the bacteriocin nisin would cause a decrease in living cells within a biofilm. In a study investigating the effects of the nisin peptide against biofilms caused by *Staphylococcus pseudintermedius* (a pathogen that affects humans and domesticated animals), it was found that the nisin A peptide could reduce, but not completely removed preformed biofilms [77]. However, the XTT assay indicated that the viable cells within the biofilm incubated with nisin A were significantly reduced compared to the untreated sample. The bioengineered derivative of nisin A used in this study showed enhanced antimicrobial effects against the biofilm-bound cells when compared to the wildtype [77].

## 3. Conclusions

The need for novel, effective, and lasting antimicrobial treatments is required now more than ever, given the impending risk to morbidity and mortality that antibiotic-resistant infections pose. With their potency at low concentrations, targeted activity, and potential for enhancement through bioengineering, bacteriocins are in the front running of antimicrobial research [8, 24, 26, 56]. Nisin, one of the very few bacteriocins currently approved for use on the market, has been applied as a preservative in food [11, 135]. It has demonstrated its capabilities not only to inhibit the growth of pathogenic food contaminating microorganisms but to extend shelf life and improve the quality of foods [16–18, 24]. Despite possessing activity against clinically relevant pathogens, no bacteriocins have been approved for medicinal use to date [8, 24, 69, 77]. It should be noted however that animal trials have confirmed the retention of bioactivity *in vivo* as well as the low toxicity levels posed by the peptides [102, 136]. In the face of declining antibiotic discoveries and escalating cases of resistant infections, it is unlikely that the current situation, where so few bacteriocins are available for widespread use, will persist.

Throughout this manuscript, we have summarised a selection of methods which can be applied to identify novel bacteriocins. While this paper focuses on wet lab experiments, it should be noted that *in silico* approaches have also been successfully applied to identify putative bacteriocin operons in bacterial genomes. However, once said operons have been identified in this manner, wet lab studies are required for the purification and characterisation of the peptide and its activity. Therefore, the methods discussed in this paper can support studies of an *in silico* nature also.

To facilitate the discovery of bacteriocins with marketable potential, reliable methods for peptide isolation and characterisation need to be developed and shared. However, complications and experimental factors that impede bioactivity during the testing process also need to be identified and discussed at length. In doing so, laboratories can be equipped with robust procedures that will identify all bacteriocin producers in a screen and that can accurately assess peptide potency in the environment it may be applied to. Failure to consider and tailor the parameters that can inhibit peptide bioactivity, e.g., improper indicator selection, poor environmental source selection, and electrostatic interactions between peptide and media, can result in poor visualisation of activity or complete oversight of antimicrobial-producing strains. As the need for novel antimicrobial treatments grows more dire, we hope the protocols and tips provided in this paper can be used to overcome such issues and accomplish the goal of discovering and characterising the bioactivity of novel bacteriocin-producing strains.

## Data Availability

No data were used to support the findings of this study.

## Supplementary Materials

Supplementary Materials Supplementary material document: protocol and diagram guide for the isolation and characterisation of bacteriocins. Figure S1: agar-based deferred antagonism assay method to screen for the presence of antimicrobial activity, created using Biorender.com. Figure S2: preparation of cell free supernatant and whole cell extract from a bacterial cell overnight culture. Both the supernatant and the whole cell extract should be assessed for bioactivity, as some peptides can remain bound to the cell surface, rather than being released into the surrounding media. If the peptide is more highly concentrated in the whole cell extract or is dispersed between both fractions, extracting peptide from both the supernatant and whole cell extract will increase yields obtained during the purification process, created using Biorender.com. Figure S3: agar-based well diffusion assay to detect antimicrobial activity. Zones of inhibition in the growth of the indicator strain indicate antimicrobial activity, created using Biorender.com. Figure S4: agarose-based radial diffusion assay to detect the presence of antimicrobial activity. Zones of inhibition in the overlay layer indicate antimicrobial action. This test can be used to detect bioactivity in samples containing low concentrations of the antimicrobial under investigation. It can also be used to detect activity against Gram-negative organisms, created using Biorender.com. Figure S5: well diffusion assay to determine protease susceptibility of antimicrobial under investigation. If the antimicrobial is sensitive to proteases, incubation with the enzyme will cause the zone of clearing in the indicator strain growth to be reduced, created using Biorender.com. Figure S6: protease spot test to determine antimicrobial sensitivity to proteolytic enzymes. If the antimicrobial under investigation is sensitive to the protease tested, the zone of clearing will be impacted. A crescent moon shaped zone, rather than a round zone of clearing, will appear in the indicator strain growth, as the antimicrobial in contact with the enzyme will be inactivated, created using Biorender.com. Figure S7: broth microdilution method to detect the minimum inhibitory concentration of an antimicrobial. The minimum inhibitory concentration is defined by the European Committee on Antimicrobial Susceptibility Testing as “the lowest concentration of the agent that completely inhibits visible growth as judged by the naked eye, disregarding a single colony or a thin haze within the area of the inoculated spot,” created using Biorender.com. Figure S8: growth curve assay to assess the impact the peptide has on bacterial culture growth. Minimum inhibitory concentrations are considered “end point assays” meaning that a singular result is obtained once the test is completed and the reaction has been stopped. Kinetic assays, like growth curves, provide insight into the effects of an antimicrobial on cell growth and death over the duration of the test at multiple time points, created using Biorender.com. Figure S9: kill curve assay. By taking samples of the peptide-exposed culture at different time points, the kill effects of the bacteriocin can be assessed, created using Biorender.com. Figure S10: analysis of biofilm inhibition on plastic surfaces using the stationary microtiter plate method. Supplementing the culture medium with 1% (w/v) D-(+) glucose has previously been found to enhance biofilm production, created using Biorender.com.

## References

[1] Riley M. A., Wertz J. E. (2002). Bacteriocins: evolution, ecology, and application. *Annual Review of Microbiology*.

[2] Blanchard A. E., Liao C., Lu T. (2016). An ecological understanding of quorum sensing-controlled bacteriocin synthesis. *Cellular and Molecular Bioengineering*.

[3] Majeed H., Gillor O., Kerr B., Riley M. A. (2011). Competitive interactions in *Escherichia coli* populations: the role of bacteriocins. *The ISME Journal*.

[4] O’Callaghan J., Buttó L. F., MacSharry J., Nally K., O’Toole P. W. (2012). Influence of adhesion and bacteriocin production by Lactobacillus salivarius on the intestinal epithelial cell transcriptional response. *Applied and Environmental Microbiology*.

[5] Cotter P. D., Hill C., Ross R. P. (2005). Bacteriocins: developing innate immunity for food. *Nature Reviews Microbiology*.

[6] Cotter P. D., Hill C., Ross R. P. (2006). What’s in a name? Class distinction for bacteriocins. *Nature Reviews Microbiology*.

[7] O’Shea E. F., O’Connor P. M., O’Sullivan O., Cotter P. D., Ross R. P., Hill C. (2013). Bactofencin A, a new type of cationic bacteriocin with unusual immunity. *MBio*.

[8] Lynch D., O’Connor P. M., Cotter P. D., Hill C., Field D., Begley M. (2019). Identification and characterisation of capidermicin, a novel bacteriocin produced by Staphylococcus capitis. *PLoS One*.

[9] Begley M., Hill C. (2010). Food safety: what can we learn from genomics?. *Annual Review of Food Science and Technology*.

[10] Sarkar C., Webster C., Gallacher J. (2018). Are exposures to ready-to-eat food environments associated with type 2 diabetes? A cross-sectional study of 347 551 UK biobank adult participants. *The Lancet Planetary Health*.

[11] U.S. Food and Drug Administration (2019). *Title 21—Food and Drugs Chapter I—Food and Drug Administration Department of Health and Human Services Subchapter B—Food for Human Consumption (Continued) Part 184—Direct Food Substances Affirmed as Generally Recognised as Safe Subpart B*.

[12] Field D., Daly K., O’Connor P. M., Cotter P. D., Hill C., Ross R. P. (2015). Efficacies of nisin A and nisin V semipurified preparations alone and in combination with plant essential oils for controlling Listeria monocytogenes. *Applied and Environmental Microbiology*.

[13] Buchanan R. L., Gorris L. G. M., Hayman M. M., Jackson T. C., Whiting R. C. (2017). A review of Listeria monocytogenes: an update on outbreaks, virulence, dose-response, ecology, and risk assessments. *Food Control*.

[14] Lappi V. R., Thimothe J., Nightingale K. K., Gall K., Scott V. N., Wiedmann M. (2004). Longitudinal studies on Listeria in smoked fish plants: impact of intervention strategies on contamination patterns. *Journal of Food Protection*.

[15] World Health Organisation (2020). Risk assessment of listeria monocytogenes in ready-to-eat foods.

[16] Economou T., Pournis N., Ntzimani A., Savvaidis I. N. (2009). Nisin-EDTA treatments and modified atmosphere packaging to increase fresh chicken meat shelf-life. *Food Chemistry*.

[17] Garavaglia J., Pinto L. M. N., Souza D. d. (2019). Natamycin and nisin to improve shelf life and minimize benzene generation in lemon soft drinks. *Food Science and Technology*.

[18] Oshima S., Hirano A., Kamikado H., Nishimura J., Kawai Y., Saito T. (2014). Nisin A extends the shelf life of high-fat chilled dairy dessert, a milk-based pudding. *Journal of Applied Microbiology*.

[19] Rodríguez J. M., Martínez M. I., Kok J. (2002). Pediocin PA-1, a wide-spectrum bacteriocin from lactic acid bacteria. *Critical Reviews in Food Science and Nutrition*.

[20] Halliwell S., Warn P., Sattar A., Derrick J. P., Upton M. (2017). A single dose of epidermicin NI01 is sufficient to eradicate MRSA from the nares of cotton rats. *Journal of Antimicrobial Chemotherapy*.

[21] Jiang H., Zou J., Cheng H., Fang J., Huang G. (2017). Purification, characterization, and mode of action of pentocin JL-1, a novel bacteriocin isolated from lactobacillus pentosus, against drug-resistant Staphylococcus aureus. *BioMed Research International*.

[22] Lee N.-K., Jin Han E., Jun Han K., Paik H.-D. (2013). Antimicrobial effect of bacteriocin KU24 produced byLactococcus lactisKU24 against methicillin-resistant Staphylococcus aureus. *Journal of Food Science*.

[23] Corr S. C., Li Y., Riedel C. U., O’Toole P. W., Hill C., Gahan C. G. M. (2007). Bacteriocin production as a mechanism for the antiinfective activity of Lactobacillus salivarius UCC118. *Proceedings of the National Academy of Sciences*.

[24] Cotter P. D., Ross R. P., Hill C. (2013). Bacteriocins—a viable alternative to antibiotics?. *Nature Reviews Microbiology*.

[25] Stern N. J., Svetoch E. A., Eruslanov B. V. (2006). Isolation of a Lactobacillus salivarius strain and purification of its bacteriocin, which is inhibitory to Campylobacter jejuni in the chicken gastrointestinal system. *Antimicrobial Agents and Chemotherapy*.

[26] Rea M. C., Sit C. S., Clayton E. (2010). Thuricin CD, a posttranslationally modified bacteriocin with a narrow spectrum of activity against Clostridium difficile. *Proceedings of the National Academy of Sciences*.

[27] Yang S.-C., Lin C.-H., Sung C. T., Fang J.-Y. (2014). Antibacterial activities of bacteriocins: application in foods and pharmaceuticals. *Frontiers in Microbiology*.

[28] Chikindas M. L., Weeks R., Drider D., Chistyakov V. A., Dicks L. M. (2018). Functions and emerging applications of bacteriocins. *Current Opinion in Biotechnology*.

[29] Chanos P., Mygind T. (2016). Co-culture-inducible bacteriocin production in lactic acid bacteria. *Applied Microbiology and Biotechnology*.

[30] Mariam S. H., Zegeye N., Tariku T., Andargie E., Endalafer N., Aseffa A. (2014). Potential of cell-free supernatants from cultures of selected lactic acid bacteria and yeast obtained from local fermented foods as inhibitors of Listeria monocytogenes, Salmonella spp. and Staphylococcus aureus. *BMC Research Notes*.

[31] Zou J., Jiang H., Cheng H., Fang J., Huang G. (2018). Strategies for screening, purification and characterization of bacteriocins. *International Journal of Biological Macromolecules*.

[32] Sugrue I., O’Connor P. M., Hill C., Stanton C., Ross R. P. (2020). Actinomyces produces defensin-like bacteriocins (actifensins) with a highly degenerate structure and broad antimicrobial activity. *Journal of Bacteriology*.

[33] Olesky P. S. (2020). Inhibition of Listeria monocytogenes by human gut bacteria. https://sword.cit.ie/scimas/4.

[34] O’Shea E. F., Gardiner G. E., O’Connor P. M., Mills S., Ross R. P., Hill C. (2009). Characterization of enterocin- and salivaricin-producing lactic acid bacteria from the mammalian gastrointestinal tract. *FEMS Microbiology Letters*.

[35] O’Sullivan J. N., Rea M. C., O’Connor P. M., Hill C., Ross R. P. (2019). Human skin microbiota is a rich source of bacteriocin-producing staphylococci that kill human pathogens. *FEMS Microbiology Ecology*.

[36] O’Sullivan J. N., O’Connor P. M., Rea M. C. (2020). Nisin J, a novel natural nisin variant, is produced by Staphylococcus capitis sourced from the human skin microbiota. *Journal of Bacteriology*.

[37] Verluyten J., Leroy F., De Vuyst L. (2004). Influence of complex nutrient source on growth of and curvacin A production by sausage isolate Lactobacillus curvatus LTH 1174. *Applied and Environmental Microbiology*.

[38] Collins F. W. J., O’Connor P. M., O’Sullivan O., Rea M. C., Hill C., Ross R. P. (2016). Formicin—a novel broad-spectrum two-component lantibiotic produced by Bacillus paralicheniformis APC 1576. *Microbiology*.

[39] Malheiros P. S., Sant’Anna V., Todorov S. D., Franco B. D. G. M. (2015). Optimization of growth and bacteriocin production by Lactobacillus sakei subsp. sakei2a. *Brazilian Journal of Microbiology*.

[40] Manzoor A., Qazi J. I., Haq I. u., Mukhtar H., Rasool A. (2017). Significantly enhanced biomass production of a novel bio-therapeutic strain Lactobacillus plantarum (AS-14) by developing low cost media cultivation strategy. *Journal of Biological Engineering*.

[41] Todorov S. D., Dicks L. M. T. (2006). Medium components effecting bacteriocin production by two strains of Lactobacillus plantarum ST414BZ and ST664BZ isolated from boza. *Biologia*.

[42] Abbasiliasi S., Tan J. S., Tengku Ibrahim T. A. (2017). Fermentation factors influencing the production of bacteriocins by lactic acid bacteria: a review. *RSC Advances*.

[43] Zendo T., Eungruttanagorn N., Fujioka S. (2005). Identification and production of a bacteriocin from Enterococcus mundtii QU 2 isolated from soybean. *Journal of Applied Microbiology*.

[44] Cladera-Olivera F., Caron G. R., Brandelli A. (2004). Bacteriocin production by Bacillus licheniformis strain P40 in cheese whey using response surface methodology. *Biochemical Engineering Journal*.

[45] Gutiérrez-Cortés C., Suarez H., Buitrago G., Nero L. A., Todorov S. D. (2018). Enhanced bacteriocin production by Pediococcus pentosaceus 147 in co-culture with Lactobacillus plantarum LE27 on cheese whey broth. *Frontiers in Microbiology*.

[46] Musatti A., Cavicchioli D., Mapelli C. (2020). From cheese whey permeate to sakacin A: a circular economy approach for the food-grade biotechnological production of an anti-Listeria bacteriocin. *Biomolecules*.

[47] Anthony T., Rajesh T., Kayalvizhi N., Gunasekaran P. (2009). Influence of medium components and fermentation conditions on the production of bacteriocin(s) by Bacillus licheniformis AnBa9. *Bioresource Technology*.

[48] Avonts L., Uytven E. V., Vuyst L. D. (2004). Cell growth and bacteriocin production of probiotic Lactobacillus strains in different media. *International Dairy Journal*.

[49] Goyal C., Malik R. K., Pradhan D. (2018). Purification and characterization of a broad spectrum bacteriocin produced by a selected Lactococcus lactis strain 63 isolated from Indian dairy products. *Journal of Food Science and Technology*.

[50] Muñoz M., Mosquera A., Alméciga-Díaz C. J., Melendez A. P., Sánchez O. F. (2012). Fructooligosaccharides metabolism and effect on bacteriocin production in Lactobacillus strains isolated from ensiled corn and molasses. *Anaerobe*.

[51] Field D., Begley M., O’Connor P. M. (2012). Bioengineered nisin A derivatives with enhanced activity against both gram positive and gram negative pathogens. *PLoS One*.

[52] McAuliffe O., Ryan M. P., Ross R. P., Hill C., Breeuwer P., Abee T. (1998). Lacticin 3147, a broad-spectrum bacteriocin which selectively dissipates the membrane potential. *Applied and Environmental Microbiology*.

[53] Ryan M. P., Rea M. C., Hill C., Ross R. P. (1996). An application in cheddar cheese manufacture for a strain of Lactococcus lactis producing a novel broad-spectrum bacteriocin. *Applied and Environmental Microbiology*.

[54] Abbasiliasi S., Ramanan R. N., Ibrahim T. A. T. (2011). Effect of medium composition and culture condition on the production of bacteriocin-like inhibitory substances (BLIS) by Lactobacillus Paracasei LA07, a strain isolated from budu. *Biotechnology & Biotechnological Equipment*.

[55] Yusuf M. A., Abdul T., Hamid T. A. (2012). Optimization of temperature and pH forthe growth and bacteriocin production of Enterococcus faecium. B3L3. *IOSR Journal of Pharmacy (IOSRPHR)*.

[56] Twomey E., Hill C., Field D., Begley M. (2020). Bioengineered nisin derivative M17Q has enhanced activity against Staphylococcus epidermidis. *Antibiotics*.

[57] Lawton E. M., Cotter P. D., Hill C., Ross R. P. (2007). Identification of a novel two-peptide lantibiotic, haloduracin, produced by the alkaliphile Bacillus halodurans C-125. *FEMS Microbiology Letters*.

[58] McClerren A. L., Cooper L. E., Quan C., Thomas P. M., Kelleher N. L., Van Der Donk W. A. (2006). Discovery and in vitro biosynthesis of haloduracin, a two-component lantibiotic. *Proceedings of the National Academy of Sciences*.

[59] Maldonado A., Ruiz-Barba J. L., Jiménez-Díaz R. (2004). Production of plantaricin NC8 by Lactobacillus plantarum NC8 is induced in the presence of different types of gram-positive bacteria. *Archives of Microbiology*.

[60] Azevedo P. O. d. S. d., Molinari F., Oliveira R. P. d. S. (2018). Importance of the agar-media in the evaluation of bacteriocin activity against the same test-microorganisms. *Brazilian Journal of Pharmaceutical Sciences*.

[61] Bevilacqua L., Ovidi M., Di Mattia E., Trovatelli L. D., Canganella F. (2003). Screening of Bifido bacterium strains isolated from human faeces for antagonistic activities against potentially bacterial pathogens. *Microbiological Research*.

[62] Janek D., Zipperer A., Kulik A., Krismer B., Peschel A. (2016). High frequency and diversity of antimicrobial activities produced by nasal Staphylococcus strains against bacterial competitors. *PLoS Pathogens*.

[63] Aznar A., Dellagi A. (2015). New insights into the role of siderophores as triggers of plant immunity: what can we learn from animals?. *Journal of Experimental Botany*.

[64] De Vuyst L., Callewaert R., Crabbé K. (1996). Primary metabolite kinetics of bacteriocin biosynthesis by Lactobacillus amylovorus and evidence for stimulation of bacteriocin production under unfavourable growth conditions. *Microbiology*.

[65] Cheng Q., Shi X., Liu Y. (2018). Production of nisin and lactic acid from corn stover through simultaneous saccharification and fermentation. *Biotechnology & Biotechnological Equipment*.

[66] Mouloud G., Daoud H., Bassem J., Laribi Atef I., Hani B. (2013). New bacteriocin from Bacillus clausii strain GM17: purification, characterization, and biological activity. *Applied Biochemistry and Biotechnology*.

[67] Bravo D., Rodríguez E., Medina M. (2009). Nisin and lacticin 481 coproduction by Lactococcus lactis strains isolated from raw ewes’ milk. *Journal of Dairy Science*.

[68] Choyam S., Lokesh D., Kempaiah B. B., Kammara R. (2015). Assessing the antimicrobial activities of ocins. *Frontiers in Microbiology*.

[69] Field D., Connor P. M. O., Cotter P. D., Hill C., Ross R. P. (2008). The generation of nisin variants with enhanced activity against specific gram-positive pathogens. *Molecular Microbiology*.

[70] O’Connor P. M., O’Shea E. F., Guinane C. M. (2015). Nisin H is a new nisin variant produced by the gut-derived strain Streptococcus hyointestinalis DPC6484. *Applied and Environmental Microbiology*.

[71] Motta A. S., Brandelli A. (2002). Characterization of an antibacterial peptide produced by Brevibacterium linens. *Journal of Applied Microbiology*.

[72] Derache C., Labas V., Aucagne V. (2009). Primary structure and antibacterial activity of chicken bone marrow-derived *β*-defensins. *Antimicrobial Agents and Chemotherapy*.

[73] Lehrer R. I., Rosenman M., Harwig S. S. S. L., Jackson R., Eisenhauer P. (1991). Ultrasensitive assays for endogenous antimicrobial polypeptides. *Journal of Immunological Methods*.

[74] Steinberg D. A., Lehrer R. I. (1997). Designer assays for antimicrobial peptides disputing the “one-size-fits-all” theory. *Antibacterial Peptide Protocols*.

[75] Healy B., Field D., O’Connor P. M., Hill C., Cotter P. D., Ross R. P. (2013). Intensive mutagenesis of the nisin hinge leads to the rational design of enhanced derivatives. *PLoS One*.

[76] Rouse S., Field D., Daly K. M. (2012). Bioengineered nisin derivatives with enhanced activity in complex matrices. *Microbial Biotechnology*.

[77] Field D., Gaudin N., Lyons F. (2015). A bioengineered nisin derivative to control biofilms of Staphylococcus pseudintermedius. *PLoS One*.

[78] Allgaier H., Jung G., Werner R. G., Schneider U., Zähner H. (1985). Elucidation of the structure of epidermin, a ribosomally synthesized, tetracyclic heterodetic polypeptide antibiotic. *Angewandte Chemie International Edition in English*.

[79] Furmanek B., Kaczorowski T., Bugalski R., Bielawski K., Bogdanowicz J., Podhajska a. A. J. (1999). Identification, characterization and purification of the lantibiotic staphylococcin T, a natural gallidermin variant. *Journal of Applied Microbiology*.

[80] Kim P. I., Sohng J. K., Sung C. (2010). Characterization and structure identification of an antimicrobial peptide, hominicin, produced by Staphylococcus hominis MBBL 2–9. *Biochemical and Biophysical Research Communications*.

[81] Sandiford S., Upton M. (2012). Identification, characterization, and recombinant expression of epidermicin NI01, a novel unmodified bacteriocin produced by Staphylococcus epidermidis that displays potent activity against staphylococci. *Antimicrobial Agents and Chemotherapy*.

[82] Parada J. L., Caron C. R., Medeiros A. B. P., Soccol C. R. (2007). Bacteriocins from lactic acid bacteria: purification, properties and use as biopreservatives. *Brazilian Archives of Biology and Technology*.

[83] Yang R., Johnson M. C., Ray B. (1992). Novel method to extract large amounts of bacteriocins from lactic acid bacteria. *Applied and Environmental Microbiology*.

[84] Åsberg D., Langborg Weinmann A., Leek T. (2017). The importance of ion-pairing in peptide purification by reversed-phase liquid chromatography. *Journal of Chromatography A*.

[85] Gaussier H., Morency H., Lavoie M. C., Subirade M. (2002). Replacement of trifluoroacetic acid with HCl in the hydrophobic purification steps of pediocin PA-1: a structural effect. *Applied and Environmental Microbiology*.

[86] Kp S., Kotnis P. V., Pv K. (2016). Production and characterization of bacteriocin produced by lactobacillus viridescence (NICM 2167). *Brazilian Archives of Biology and Technology*.

[87] Ge J., Sun Y., Xin X., Wang Y., Ping W. (2016). Purification and partial characterization of a novel bacteriocin synthesized by Lactobacillus paracasei HD1-7 isolated from Chinese sauerkraut juice. *Scientific Reports*.

[88] Uteng M., Hauge H. H., Brondz I., Nissen-Meyer J., Fimland G. (2002). Rapid two-step procedure for large-scale purification of pediocin-like bacteriocins and other cationic antimicrobial peptides from complex culture medium. *Applied and Environmental Microbiology*.

[89] Ishibashi N., Himeno K., Fujita K. (2012). Purification and characterization of multiple bacteriocins and an inducing peptide produced by Enterococcus faecium NKR-5-3 from Thai fermented fish. *Bioscience, Biotechnology, and Biochemistry*.

[90] Josic D., Kovac S. (2010 Aug). Reversed‐phase high performance liquid chromatography of proteins. *Current Protocols in Protein Science*.

[91] Abts A., Mavaro A., Stindt J. (2011). Easy and rapid purification of highly active nisin. *International Journal of Peptides*.

[92] Rezaei Javan R., van Tonder A. J., King J. P., Harrold C. L., Brueggemann A. B. (2018). Genome sequencing reveals a large and diverse repertoire of antimicrobial peptides. *Frontiers in Microbiology*.

[93] Walsh C. J., Guinane C. M., Hill C., Ross R. P., O’Toole P. W., Cotter P. D. (2015). In silico identification of bacteriocin gene clusters in the gastrointestinal tract, based on the human microbiome project’s reference genome database. *BMC Microbiology*.

[94] Wang H., Fewer D. P., Sivonen K. (2011). Genome mining demonstrates the widespread occurrence of gene clusters encoding bacteriocins in cyanobacteria. *PLoS One*.

[95] Sandiford S. K. (2017). Genome database mining for the discovery of novel lantibiotics. *Expert Opinion on Drug Discovery*.

[96] Lajis A. F. B. (2020). Biomanufacturing process for the production of bacteriocins from Bacillaceae family. *Bioresources and Bioprocessing*.

[97] Yang E., Fan L., Yan J. (2018). Influence of culture media, pH and temperature on growth and bacteriocin production of bacteriocinogenic lactic acid bacteria. *AMB Express*.

[98] Delves-broughton J. (1990). Nisin and its application as a food preservative. *International Journal of Dairy Technology*.

[99] Younes M., Younes M., Aggett P. (2017). Safety of nisin (E 234) as a food additive in the light of new toxicological data and the proposed extension of use. *EFSA Journal*.

[100] Gardiner G. E., Rea M. C., O’Riordan B. (2007). Fate of the two-component lantibiotic lacticin 3147 in the gastrointestinal tract. *Applied and Environmental Microbiology*.

[101] Piper C., Casey P. G., Hill C., Cotter P. D., Ross R. P. (2012). The lantibiotic lacticin 3147 prevents systemic spread of Staphylococcus aureus in a murine infection model. *International Journal of Microbiology*.

[102] Campion A., Casey P. G., Field D., Cotter P. D., Hill C., Ross R. P. (2013). In vivo activity of nisin A and nisin V against Listeria monocytogenes in mice. *BMC Microbiology*.

[103] Molloy E. M., Field D., Connor P. M. O., Cotter P. D., Hill C., Ross R. P. (2013). Saturation mutagenesis of lysine 12 leads to the identification of derivatives of nisin A with enhanced antimicrobial activity. *PLoS One*.

[104] European Committee for Antimicrobial Susceptibility Testing (EUCAST) of the European Society of Clinical Microbiology and Infectious Dieases (ESCMID) (2000). Determination of minimum inhibitory concentrations (MICs) of antibacterial agents by agar dilution. *Clinical Microbiology and Infection*.

[105] Field D., Quigley L., O’Connor P. M. (2010). Studies with bioengineered nisin peptides highlight the broad-spectrum potency of nisin V. *Microbial Biotechnology*.

[106] Zhang J., Yang Y., Yang H. (2018). Purification and partial characterization of bacteriocin lac-B23, a novel bacteriocin production by Lactobacillus plantarum J23, isolated from Chinese traditional fermented milk. *Frontiers in Microbiology*.

[107] Field D., Blake T., Mathur H. (2019). Bioengineering nisin to overcome the nisin resistance protein. *Molecular Microbiology*.

[108] Hanson H., Whitfield Y., Lee C. (2019). Listeria monocytogenes associated with pasteurized chocolate milk, Ontario, Canada. *Emerging Infectious Diseases*.

[109] Cody S. H., Glynn M. K., Farrar J. A. (1999). An outbreak of *Escherichia coli* O157:H7 infection from unpasteurized commercial apple juice. *Annals of Internal Medicine*.

[110] FAO (2006). Enterobacter sakazakii and other microorganisms in powdered infant formula. *Microbiol Risk Assessment*.

[111] Boelaert F., Van der Stede Y., Stoicescu A. (2018). The European Union summary report on trends and sources of zoonoses, zoonotic agents and food‐borne outbreaks in 2017. *EFSA Journal*.

[112] Begley M., Cotter P. D., Hill C., Ross R. P. (2010). Glutamate decarboxylase-mediated nisin resistance in Listeria monocytogenes. *Applied and Environmental Microbiology*.

[113] Spanu C., Scarano C., Ibba M., Pala C., Spanu V., De Santis E. P. L. (2014). Microbiological challenge testing for Listeria monocytogenes in ready-to-eat food: a practical approach. *Italian Journal of Food Safety*.

[114] Campion A., Morrissey R., Field D., Cotter P. D., Hill C., Ross R. P. (2017). Use of enhanced nisin derivatives in combination with food-grade oils or citric acid to control cronobacter sakazakii and *Escherichia coli* O157:H7. *Food Microbiology*.

[115] Davidson P. M., Critzer F. J., Taylor T. M. (2013). Naturally occurring antimicrobials for minimally processed foods. *Annual Review of Food Science and Technology*.

[116] Tremblay Y. D., Lévesque C., Segers R. P., Jacques M. (2013). Method to grow Actinobacillus pleuropneumoniae biofilm on a biotic surface. *BMC Veterinary Research*.

[117] Bressler D., Balzer M., Dannehl A., Flemming H.-C., Wingender J. (2009). Persistence of Pseudomonas aeruginosa in drinking-water biofilms on elastomeric material. *Water Supply*.

[118] Vestby L. K., Møretrø T., Langsrud S., Heir E., Nesse L. L. (2009). Biofilm forming abilities of Salmonella are correlated with persistence in fish meal- and feed factories. *BMC Veterinary Research*.

[119] Anderl J. N., Franklin M. J., Stewart P. S. (2000). Role of antibiotic penetration limitation in Klebsiella pneumoniae biofilm resistance to ampicillin and ciprofloxacin. *Antimicrobial Agents and Chemotherapy*.

[120] Cherif-Antar A., Moussa-Boudjemâa B., Didouh N., Medjahdi K., Mayo B., Flórez A. B. (2016). Diversity and biofilm-forming capability of bacteria recovered from stainless steel pipes of a milk-processing dairy plant. *Dairy Science & Technology*.

[121] Latorre A. A., Van Kessel J. S., Karns J. S. (2010). Biofilm in milking equipment on a dairy farm as a potential source of bulk tank milk contamination with Listeria monocytogenes. *Journal of Dairy Science*.

[122] Wijman J. G. E., De Leeuw P. P. L. A., Moezelaar R., Zwietering M. H., Abee T. (2007). Air-liquid interface biofilms of Bacillus cereus: formation, sporulation, and dispersion. *Applied and Environmental Microbiology*.

[123] Centorame P., D’Angelo A. R., Di Simone F. (2017). Listeria monocytogenes biofilm production on food packaging materials submitted to physical treatment. *Italian Journal of Food Safety*.

[124] Dewanti R., Wong A. C. L. (1995). Influence of culture conditions on biofilm formation by *Escherichia coli* O157:H7. *International Journal of Food Microbiology*.

[125] Khelissa S. O., Jama C., Abdallah M., Boukherroub R., Faille C., Chihib N.-E. (2017). Effect of incubation duration, growth temperature, and abiotic surface type on cell surface properties, adhesion and pathogenicity of biofilm-detached Staphylococcus aureus cells. *AMB Express*.

[126] Jensen E. T., Kharazmi A., Lam K., Costerton J. W., Høiby N. (1990). Human polymorphonuclear leukocyte response to Pseudomonas aeruginosa grown in biofilms. *Infection and Immunity*.

[127] Sandle T. (2015). Characterizing the microbiota of a pharmaceutical water system—a metadata study. *SOJ Microbiology & Infectious Diseases*.

[128] Field D., O’Connor R., Cotter P. D., Ross R. P., Hill C. (2016). In vitro activities of nisin and nisin derivatives alone and in combination with antibiotics against Staphylococcus biofilms. *Frontiers in Microbiology*.

[129] Field D., Seisling N., Cotter P. D., Ross R. P., Hill C. (2016). Synergistic nisin-polymyxin combinations for the control of Pseudomonas biofilm formation. *Frontiers in Microbiology*.

[130] Mathur H., Field D., Rea M. C., Cotter P. D., Hill C., Ross R. P. (2018). Fighting biofilms with lantibiotics and other groups of bacteriocins. *NPJ Biofilms and Microbiomes*.

[131] Rumbaugh K. P., Sauer K. (2020). Biofilm dispersion. *Nature Reviews Microbiology*.

[132] Christensen G. D., Simpson W. A., Younger J. J. (1985). Adherence of coagulase-negative staphylococci to plastic tissue culture plates: a quantitative model for the adherence of staphylococci to medical devices. *Journal of Clinical Microbiology*.

[133] Mathur T., Singhal S., Khan S., Upadhyay D., Fatma T., Rattan A. (2006). Detection of biofilm formation among the clinical isolates of staphylococci: an evaluation of three different screening methods. *Indian Journal of Medical Microbiology*.

[134] Lynch D., Hill C., Field D., Begley M. (2021). Inhibition of Listeria monocytogenes by the Staphylococcus capitis—derived bacteriocin capidermicin. *Food Microbiology*.

[135] European Food Safety Authority (2006). Opinion of the scientific panel on food additives, flavourings, processing aids and materials in contact with food on a request from the commission related to the use of nisin (E 234) as a food additive. http://www.efsa.eu.int/science/afc/afc_opinions/catindex_en.html.

[136] Bower C. K., Parker J. E., Higgins A. Z. (2002). Protein antimicrobial barriers to bacterial adhesion: in vitro and in vivo evaluation of nisin-treated implantable materials. *Colloids and Surfaces B: Biointerfaces*.

