# Supplementary material for: Recipe for Success: Suggestions and Recommendations for the Isolation and Characterisation of Bacteriocins
Source: Int J Microbiol. 2021 Jun 17;2021:9990635. doi: 10.1155/2021/9990635 (PMC8249226; doi:10.1155/2021/9990635)
Supplement: Supplementary Materials — Supplementary material document: protocol and diagram guide for the isolation and characterisation of bacteriocins. Figure S1: agar-based deferred antagonism assay method to screen for the presence of antimicrobial activity, created using Biorender.com. Figure S2: preparation of cell free supernatant and whole cell extract from a bacterial cell overnight culture. Both the supernatant and the whole cell extract should be assessed for bioactivity, as some peptides can remain bound to the cell surface, rather than being released into the surrounding media. If the peptide is more highly concentrated in the whole cell extract or is dispersed between both fractions, extracting peptide from both the supernatant and whole cell extract will increase yields obtained during the purification process, created using Biorender.com. Figure S3: agar-based well diffusion assay to detect antimicrobial activity. Zones of inhibition in the growth of the indicator strain indicate antimicrobial activity, created using Biorender.com. Figure S4: agarose-based radial diffusion assay to detect the presence of antimicrobial activity. Zones of inhibition in the overlay layer indicate antimicrobial action. This test can be used to detect bioactivity in samples containing low concentrations of the antimicrobial under investigation. It can also be used to detect activity against Gram-negative organisms, created using Biorender.com. Figure S5: well diffusion assay to determine protease susceptibility of antimicrobial under investigation. If the antimicrobial is sensitive to proteases, incubation with the enzyme will cause the zone of clearing in the indicator strain growth to be reduced, created using Biorender.com. Figure S6: protease spot test to determine antimicrobial sensitivity to proteolytic enzymes. If the antimicrobial under investigation is sensitive to the protease tested, the zone of clearing will be impacted. A crescent moon shaped zone, rather than a round zone of clearing, will appea [file 9990635.f1.docx]

**Supplementary Material Document:** Protocol and diagram guide for the isolation and characterisation of bacteriocins.

|  | **Table S1: Guidelines for growth conditions of indicator strains*** | | Page **2** of **49** |
| --- | --- | --- | --- |
| **Strain Name** | **Important Characteristics** | **Media type*** | **Incubation conditions** |
| *Lactococcus lactis* NZ9700 | Nisin producing organism | GM17 broth and agar | 30^o^C, 16 – 18 hours |
| *Lactococcus lactis* spp. *cremoris*  HP | Antimicrobial sensitive strain | GM17 broth and agar | 30^o^C, 16 – 18 hours |
| *Micrococcus luteus* | Antimicrobial sensitive strain | BHI broth and agar | 37^o^C, 16 – 18 hours |
| *Listeria innocua* | Representative of food contaminating organisms. Genetically similar to serious food pathogen, *L. monocytogenes* | BHI broth and agar | 37^o^C, 16 – 18 hours |
| *Listeria monocytogenes* | Nisin sensitive indicator | BHI broth and agar | 37^o^C, 16 – 18 hours |
| *Staphylococcus aureus* | Strain representative of health care pathogen | BHI broth and agar | 37^o^C, 16 – 18 hours |
| *Staphylococcus epidermidis* | Biofilm forming organism representative of health care  pathogen | TS broth and agar | 37^o^C, 16 – 18 hours |
| *Escherichia coli* O157:H7 | Gram negative indicator strain | Luria-Bertani broth and agar | 37^o^C, 16 – 18 hours |
| *Salmonella enterica* serovar Typhimurium UK1 | Gram negative indicator strain | Luria-Bertani broth and agar | 37^o^C, 16 – 18 hours |

* Media types: (a) M17 agar and broth supplemented with 0.5% glucose, (b) Brain Heart Infusion broth and agar, (c) TS: Tryptone soy broth and agar, supplemented with 1% glucose during biofilm formation.

**Table S2: List of protocols and accompanying figures in the order they appear the Supplementary Material Document: Protocol and diagram guide for the isolation and characterisation of bacteriocins.**

| **Protocol Number** | **Protocol Title** | **Accompanying Figure** |
| --- | --- | --- |
| **S1** | Preparation of microbial culture medium and overnight cultures | N/A |
| **S2(A)** | Deferred antagonism assays for the identification of antimicrobial activity | Figure S1 |
| **S2(B)** | High through put deferred antagonism assays for the identification of antimicrobial activity. | N/A |
| **S3** | Preparation of cell free supernatant and whole cell extract. | Figure S2 |
| **S4** | Agar based well diffusion assays. | Figure S3 |
| **S5** | Agarose-based radial diffusion assay. | Figure S4 |
| **S6** | Purification of bacteriocin peptides | N/A |
| **S7(A)** | Stability assay - incubation with protease enzymes | Figure S5 |
| **S7(B)** | Susceptibility assays - crescent moon protease assay | Figure S6 |
| **S7(C)** | Susceptibility assay – pH treatment | N/A |
| **S7(D)** | Susceptibility assay – temperature treatment | N/A |
| **S8** | Minimum inhibitory concentration assay | Figure S7 |
| **S9** | Growth assay | Figure S8 |
| **S10** | Kill curve assay | Figure S9 |
| **S11(A)** | Model food trials with a liquid food sample | N/A |
| **S11(B)** | Model food trials with a solid food sample | N/A |
| **S12** | Inhibition of biofilm on plastic surfaces | Figure S10 |
| **S13** | Removal of pre-formed biofilm on plastic microtiter plates | N/A |

**Protocol S1: Preparation of microbial culture medium and overnight cultures**

# Preparing culture medium

1. Prepare broth which is relevant to the isolated strain or the indicator strain, as per the manufacturers’ instructions, i.e. add the correct quantity of the dehydrated media powder to sterile, deionised water.
2. Sterilise the media by autoclaving at 121^o^C, for 15 minutes at 15 psi, unless otherwise specified.
3. Cool the sterile media to 50^o^C using a calibrated water bath or incubator.
4. Distribute the cooled media to sterile containers, e.g. falcon tubes (Sarstedt, order no. 62.547.254), test tubes (Fisher Scientific, product no. 15802285), Duran bottles (DURAN, cat no. 21801175) or universal tubes (Sarstedt, order no. 63.9922.254) at required volumes.
5. Aseptically inoculate the relevant broth type with a single, bacterial colony from a streak plate or a serial dilution spread plate, (less than 30 hours old).
6. Incubate the cultures “overnight”, (for 16 – 18 hours), at a relevant temperature.
7. When selecting a broth type, consider the strains type as some species have specific nutritional requirements, e.g. Tryptic soy broth for the growth of *Staphylococci*. 10mL of the relevant broth is a suitable volume for an initial overnight culture, unless otherwise specified. Colonies used to inoculate the media should be fresh

***Tips***:

**a.** Prepare all media on the day to ensure that it is fresh and of the best quality.

**Protocol S2(A): Deferred antagonism assays for the identification of antimicrobial activity.**

**See *Figure S1* for protocol diagram.**

# Preparing culture plates

1. Prepare a culture of the isolated strain under investigation as per **Protocol S1**, at a final volume of 10mL.
2. Following incubation transfer a 20µL volume of the overnight culture to the centre of a fresh agar plate of the relevant media type.
3. Dry the spot cultures in a sterile biological safety cabinet. Once dry, invert plates and incubate for 16 – 18 hours at the relevant temperature for the isolate.

**Preparing indicator strain**

1. Prepare a culture of the indicator strain as per **Protocol S1**, at a final volume of 10mL.

# Overlaying plates to determine antimicrobial activity

1. Following incubation, remove the lids of the spot plates within the UV box and expose the spotted cultures, (cultures facing the bulbs), to the maximum wavelength for 30 minutes.
2. Prepare 0.75% w/v “sloppy” agar, divide into 10mL volumes, and bring to a temperature of 50ºC using an incubator or water bath.
3. Inoculate each 10mL volume with 100µL of indicator strain overnight culture, (1% inoculum).
4. Pour the seeded agar over the UV treated plate in a controlled manner as not to dislodge the cultures.
5. Allow the agar to solidify then incubate upright at the relevant temperature for the indicator strain for 16 – 18 hours. Following incubation, inspect the plates for zones of clearing in the overlaid organism. Measure the zones with a Vernier Callipers across the width of the zone.

Perform in triplicate using three biological repeats, i.e. three separate overnight cultures of the bacteriocin producing strain, to determine the standard deviation and confirm antimicrobial action.

***Tips:***

1. Prepare all media on the day to ensure that it is fresh and of the best quality.
2. Sterilise the UV box prior to use by wiping down all internal surfaces with 70% isopropanol.
3. Run the UV box for 10 minutes at max strength to warm the bulbs prior to use.
4. UV Crosslinkers can also be used to treat the spot cultures, e.g. CL-1000 Ultraviolet Crosslinker. As with UV boxes, sterilise by wiping down all internal surfaces with 70% isopropanol. Run the Crosslinker for 10 minutes at the maximum strength to warm the bulbs prior to use. Remove lids and place the plates inside the crosslinker, (cultures facing the bulbs), and treat for 30 minutes at the maximum strength.
5. Remove and replace lids within the sterile confines of the UV box or UV Crosslinker as to avoid contaminating the treated cultures.
6. Keep the volumes of 0.75% w/v “sloppy” agar in a water bath or incubator right up until the time they are required, as agar solidifies quickly and can form clumps when poured.

Page **6** of **49**


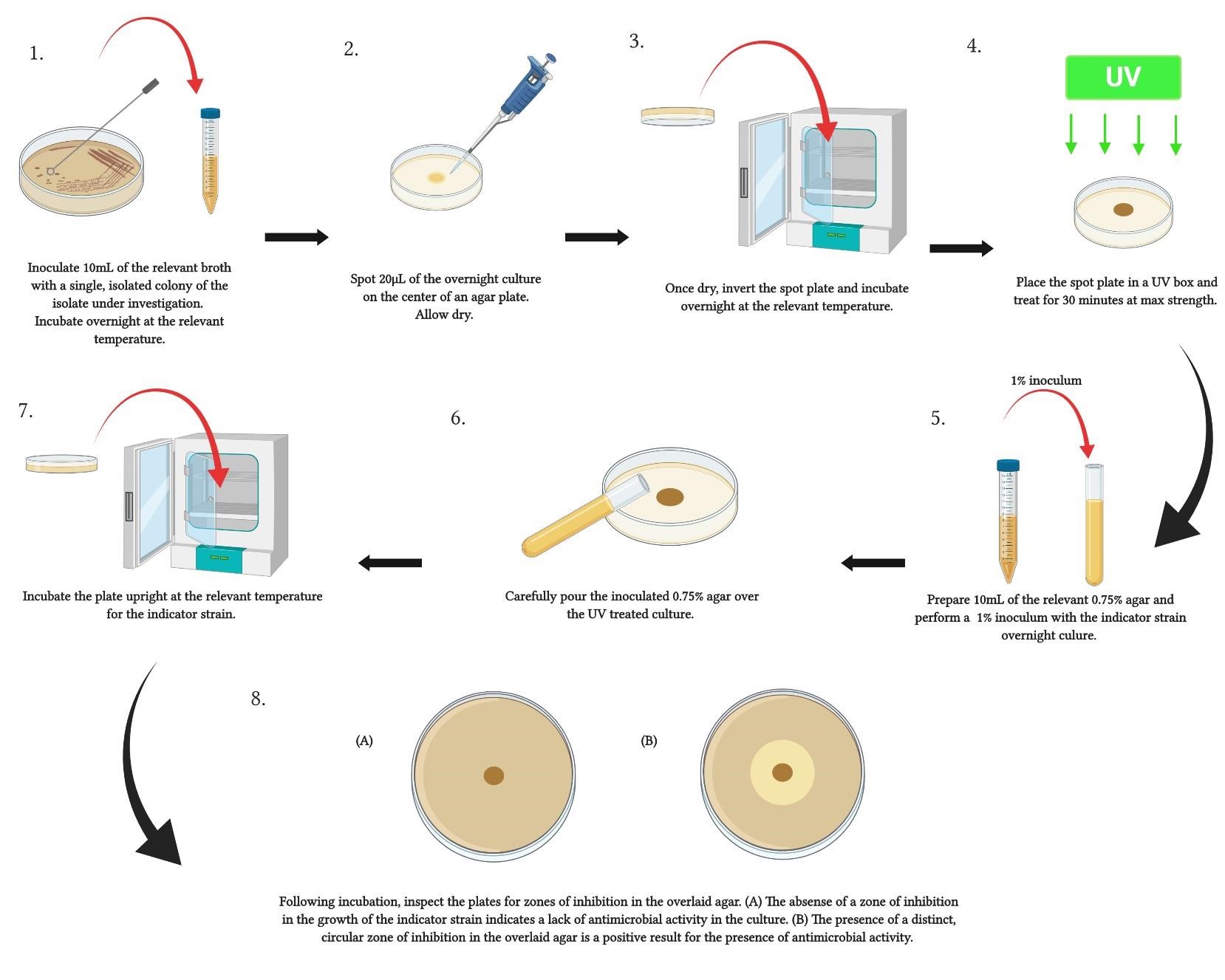


**Figure S1: Agar-based deferred antagonism assay method to screen for the presence of antimicrobial activity. Created using Biorender.com.**

**Protocol S2(B): High through put deferred antagonism assays for the identification of antimicrobial activity.**

**Preparation of cultures and agar for high through put deferred antagonism assay.**

1. Prepare a culture of the isolated strain under investigation as per **Protocol S1**, at a final volume of 10mL.
2. Following incubation aseptically transfer 200µL volumes of each overnight culture to individual wells of a sterile 96 well microtiter plate, (Round well, flat bottomed microtiter plate, with a lid; Sarstedt, order no. 82.1582.001).
3. Prepare the relevant agar type for the isolate cultures, then pour into large square petri dishes (Greiner, cat no. 688102G), (120x120x17mm).
4. Sterilise a 96 well replicator by dipping in 70% isopropanol and passing through a Bunsen flame (Boekel Scientific, cat no. 140500-140501-140384).
5. Dip the pins of the replicator to the wells containing overnight culture of the isolate strains.
6. Transfer the culture to surface of the agar.
7. Allow spotted cultures to dry in a sterile biological safety cabinet.
8. Once dried, invert spot plates and incubate overnight at the relevant temperature for isolates.

**Preparing indicator strain**

1. Prepare a culture of the indicator strain as per **Protocol S1**, at a final volume of 10mL.

# Overlaying plates to determine antimicrobial activity

1. Following incubation, remove the lids of the spot plates inside the UV box and expose the spotted cultures, (culture side facing the bulbs), to the maximum wavelength for 30 minutes.
2. Prepare 0.75% w/v “sloppy” agar, divide into 20mL volumes, and bring to a temperature of 50ºC using an incubator or water bath.
3. Inoculate each 20mL volume with 40µL of indicator strain culture, (2% inoculum).
4. Aseptically pour the seeded agar over the UV treated spot cultures in a controlled manner as not to dislodge the cultures.
5. Allow the agar to set then incubate upright at the relevant temperature for the indicator strain for 16 – 18 hours. Following incubation, inspect the plates for zones of clearing

in the overlaid organism. Measure the zones with a Vernier Callipers across the width of the zone.

Perform in triplicate using three biological repeats, i.e. three separate overnight cultures of the bacteriocin producing strain, to determine the standard deviation and confirm antimicrobial action.

***Tips:***

1. Prepare all media on the day to ensure that it is fresh and of the best quality.
2. When transferring cultures to the 96 well plate, only every second well should be filled.

This permits space between plated cultures to prevent merging zones.

1. Sterilise the UV box prior to use by wiping all internal surfaces with 70% isopropanol.
2. Run the UV box for 10 minutes at max strength to warm the bulbs prior to use.
3. UV Crosslinkers can also be used to treat the spot cultures, e.g. CL-1000 Ultraviolet Crosslinker. As with UV boxes, sterilise by wiping down all internal surfaces prior to use with 70% isopropanol. Run the Crosslinker for 10 minutes at the maximum strength to warm the bulbs prior to use. Remove lids and place the plates inside the crosslinker, (cultures facing the bulbs), and treat for 30 minutes at the maximum strength.
4. The lids of plates should be removed and replaced within the UV box or UV Crosslinker to avoid contaminating the treated cultures.
5. Keep the volumes of 0.75% w/v “sloppy” agar in a water bath or incubator right up until the time they are required, as agar sets quickly and can form clumps when poured.
6. 96 well plates can be used to create stocks that facilitate high through put screening. 80% glycerol can be added at a 1:1 ratio, (100µL of bacterial culture and 100µL of 80% glycerol), to the individual cultures in the wells with a multichannel pipette (Eppendorf, 12-channel variable pipette, cat no. 3125000028). Ensure that pipette tips are changed after contact with each culture to prevent contamination. Plates can then be safely kept at -80^o^C for long term storage. Seal the plates with parafilm to create an airtight environment within the plate. When removing from storage, thaw plates on a bed of ice to reduce thermal shock.

**Protocol S3: Preparation of cell free supernatant and whole cell extract.**

**See *Figure S2* for protocol diagram.**

**Preparation of cultures.**

1. Prepare a culture of the isolated strain under investigation, at a final volume of 50mL, as per **Protocol S1**.

**S3(a) Preparation of cell free supernatant.**

1. Centrifuge isolate overnight cultures at 7000 x *g* for 20 minutes at 4ºC to pellet the cells (IEC CL30R, Thermo Scientific).
2. Using an electric, automated pipette gun (Fisher Scientific, cat no. 15249805) and a sterile 25mL serological pipette tip (Sarstedt, order no. 86.1685.001), aseptically separate the supernatant from the cell pellet.
3. Transfer the cell free supernatant to a sterile falcon tube (Sarstedt, order no.

62.547.254).

**S3(b) Preparation of the whole cell extract.**

1. Resuspend the cell pellet obtained in Step 3 in a 5mL volume of 70% isopropanol, 0.1% trifluoracetic acid (Sigma Aldrich, product no. 302031-100ML) and water. Transfer the resuspended pellet to a universal tube (Sarstedt, order no. 63.9922.254).
2. Place the universal tubes in a shaking incubator, (Heidolph, product no. 544-11200-00), and set shaking at 1,000RPM for three hours, at room temperature.
3. After three hours agitation, centrifuge the solution at 6000 x *g* for fifteen minutes at 4ºC to pellet cellular debris, (IEC CL30R, Thermo Scientific).
4. Using an electric, automated pipette gun (Fisher Scientific, cat no. 15249805) and a 10mL serological pipette tip (Fisher Scientific, cat no. 10084450), separate the supernatant i.e. the whole cell extract from the pelleted cellular debris.
5. Transfer the whole cell extract to a fresh, sterile falcon tube (Sarstedt, order no.

62.547.254).

***Tips:***

1. All media should be prepared on the day of use to ensure it is of the best quality.
2. The cell free supernatant and whole cell extract should be used on the day they are prepared. This ensures the solutions are fresh and that no bioactivity has been lost.
3. Once centrifugation is complete, transfer the supernatant as quickly as possible to a separate sterile container from the pellet or cellular debris. This prevents the pellet or debris from becoming resuspended in the supernatant.
4. Supernatants can be filter sterilised using a 0.45µm pore filter, (Sarstedt, order no.

83.1826), to ensure that all cells and debris have been removed.

1. Gently vortex the supernatant and whole cell extract before use.


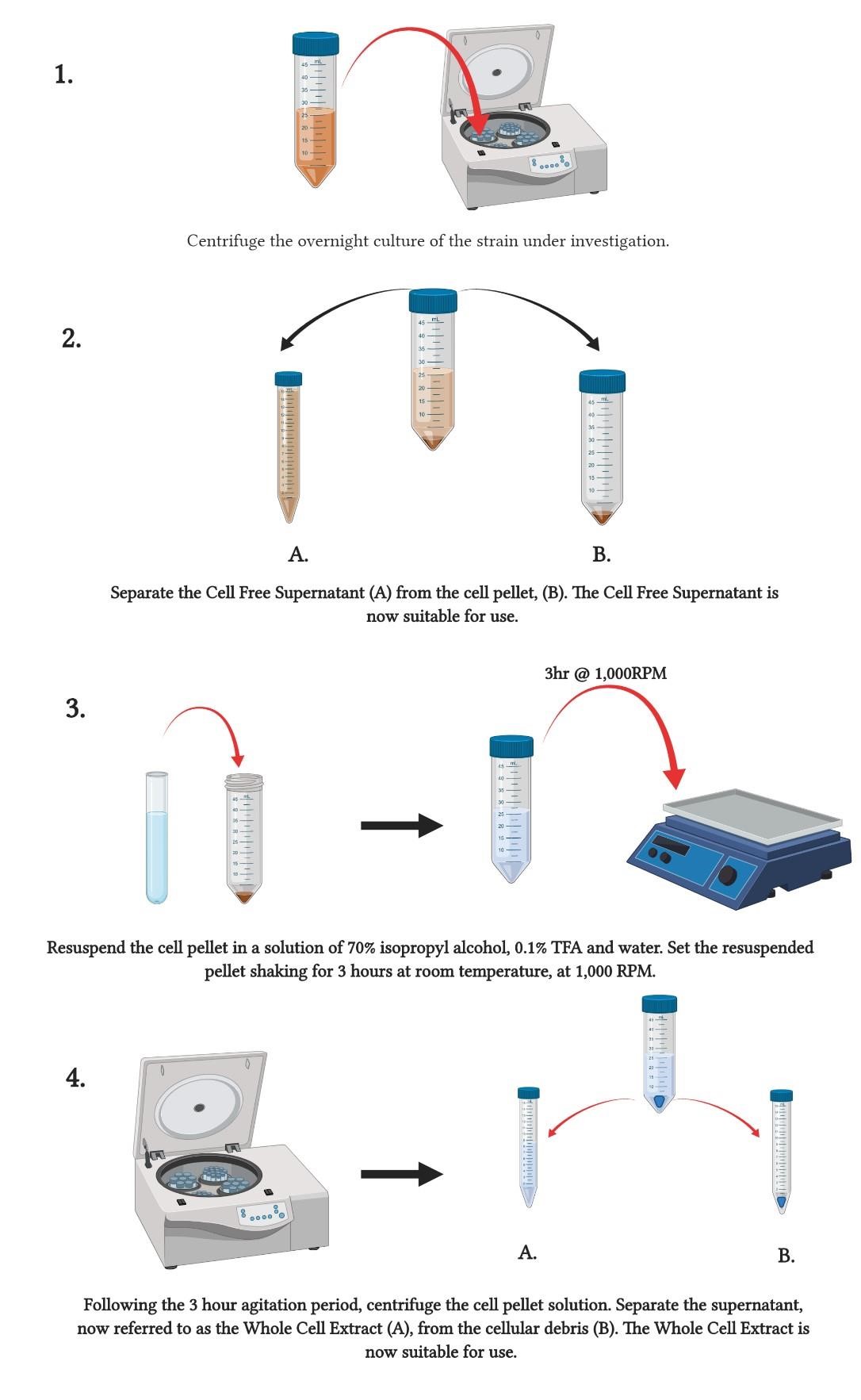


**Figure S2: Preparation of cell free supernatant and whole cell extract from a bacterial cell overnight culture. Both the supernatant and the whole cell extract should be assessed for bioactivity, as some peptides can remain bound to the cell surface, rather than being released into the surrounding media. If the peptide is more highly concentrated in the whole cell extract or is dispersed between both fractions, extracting peptide from both the supernatant and whole cell extract will increase yields obtained during the purification process. Created using Biorender.com.**

**Protocol S4: Agar based well diffusion assays.**

**See *Figure S3* for protocol diagram.**

**Preparing indicator strain**

1. Prepare a culture of the indicator strain as per **Protocol S1**, at a final volume of 10mL.

# Performing well diffusion assays

1. Prepare the relevant agar for the indicator strain and cool to 50^o^C with a calibrated water bath or incubator.
2. Using an electric, automated pipette gun (Fisher Scientific, cat no. 15249805) and a sterile 25mL pipette tip (Sarstedt, order no. 86.1685.001), aseptically transfer 20mL volumes of molten agar to sterile falcon tubes (Sarstedt, order no. 62.547.254).
3. Inoculate each 20mL volume with 200µL of indicator strain culture, (1% inoculum).
4. Pour the seeded agar into sterile petri dishes and allow to set.
5. Take a glass Pasteur pipette (VWR International Limited, cat no. 612-3813), (6mm diameter), and dip the end briefly in 70% isopropanol before passing through a Bunsen flame to sterilise.
6. Use the sterile rod to punch several wells in the agar before removing the agar plugs by picking with a sterile pipette tip and discard.
7. Transfer volumes of each test sample to individual wells in agar.

# Determining antimicrobial activity

1. Incubate the plates overnight, upright at the relevant temperature for the indicator strain.
2. Following incubation, measure the zones of inhibition around the wells in the agar with a Vernier callipers.

Perform in triplicate using three biological repeats, i.e. three separate overnight cultures of the bacteriocin producing strain, to determine the standard deviation and confirm antimicrobial action.

**Tip*s*:**

1. Agar well diffusion assays can be used to assess the potential bioactivity of purified peptides, HPLC fractions, cell free supernatants and the whole cell extract of a culture.
2. Keep the volumes of agar in a water bath or incubator right up until the time they are required, as agar solidifies quickly and can form clumps when poured.
3. Before punching holes in the agar, allow the rod to cool within range of the Bunsen flame to maintain sterility. Doing so makes it easier to remove the agar plugs, as the heated rod can liquify the media and prevent them being lifted from the well.
4. When removing the agar plugs with a pipette tip, skewer the plugs at an angle and lift out to avoid making tears in the surrounding media.
5. If performing well diffusion assays on the CFS of a culture, neutralise the solution to pH 7 to ensure that acid production during growth does not contribute to the zone size.


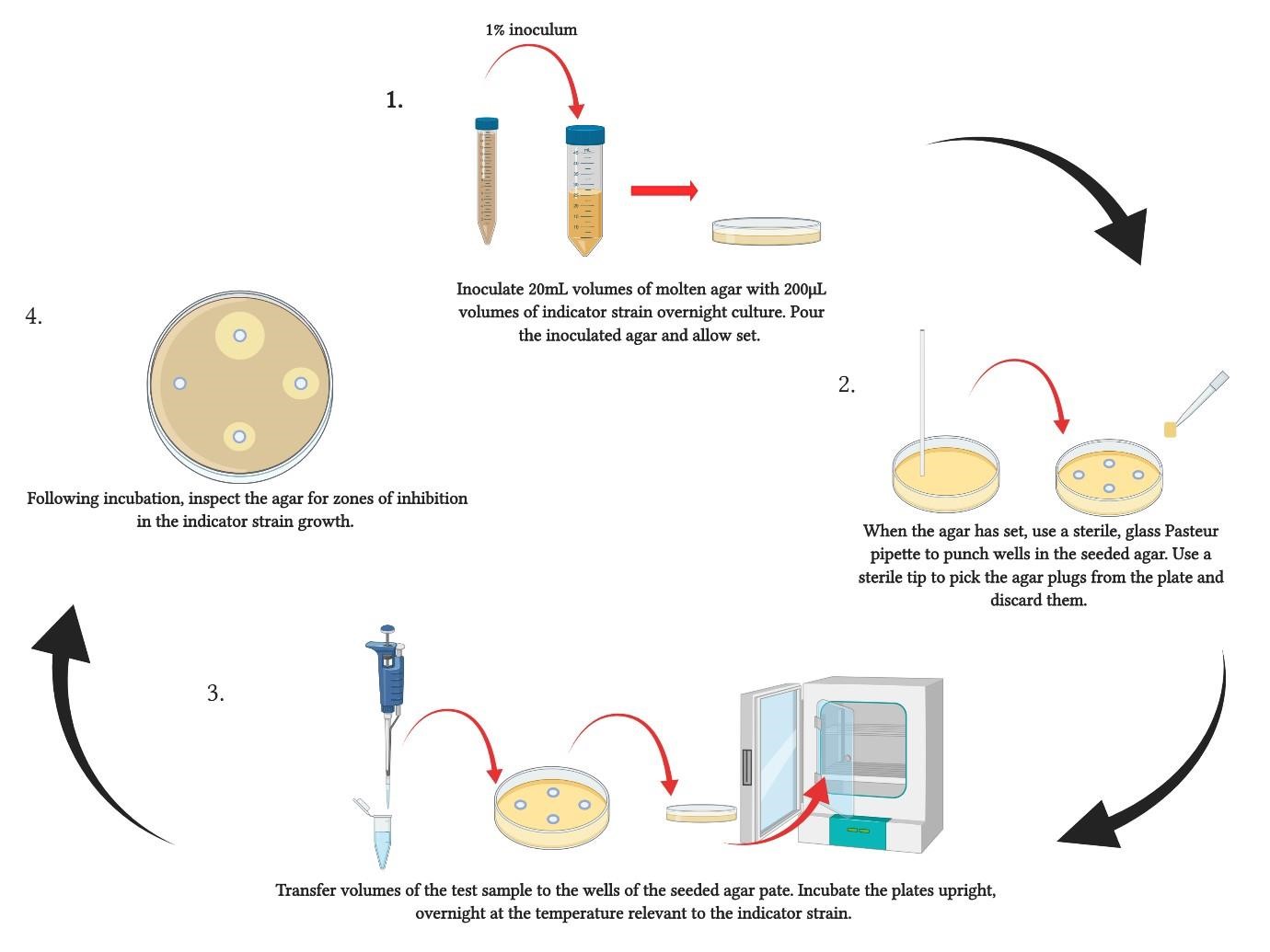


**Figure S3: Agar-based well diffusion assay to detect antimicrobial activity. Zones of inhibition in the growth of the indicator strain indicate antimicrobial activity. Created using Biorender.com.**

**Protocol S5: Agarose-based radial diffusion assay.**

**See *Figure S4* for protocol diagram.**

# Preparing cultures for plating

1. Prepare a culture of the isolated strain under investigation, as per **Protocol S1** at a final volume of 10mL.
2. Prepare a culture of the indicator strain as per **Protocol S1**, at a final volume of 10mL.

# Preparing plates to determine antimicrobial action

1. Prepare the underlay by combining 10 mM sodium phosphate buffer (pH 7.4) (Sigma Aldrich, product no. P5244-100ML) and 1% (w/v) agarose (Sigma Aldrich, product no. A9539-100G). Autoclave to ensure sterility.
2. Using an electric, automated pipette gun (Fisher Scientific, cat no. 15249805) and a sterile 25mL pipette tip (Sarstedt, order no. 86.1685.001), aseptically transfer 15mL volumes of underlay solution to sterile falcon tubes (Sarstedt, order no. 62.547.254) and cool to 50ºC using a water bath or incubator.
3. Inoculate each 15mL volume with 200µL of indicator strain culture, (1% inoculum).
4. Pour the inoculated underlay into petri dishes and allow set.
5. Take a glass Pasteur pipette (VWR International Limited, cat no. 612-3813), (6mm diameter), and dip the top end briefly in 70% isopropanol before passing through a Bunsen flame to sterilise.
6. Use the sterile rod to punch several holes in the agar before removing the agar plugs by picking with a sterile pipette tip and discard.
7. Transfer volumes of each test sample to individual wells in agar.
8. Incubate plates upright at the relevant temperature for the indicator for three hours.

# Overlaying plates and determining antimicrobial action

1. During the incubation period, prepare the relevant broth at double strength concentration, i.e. twice the recommended weight per volume, supplemented with 1% (w/v) agarose. Autoclave to ensure sterility.
2. Overlay the agarose well plates with double strength broth supplemented with 1% agarose.
3. Incubate upright overnight at the relevant temperature for the indicator strain.
4. Following incubation, measuring any zones of clearing that appear in the overlaid agar with a Vernier callipers.

Perform in triplicate using three biological repeats, i.e. three separate overnight cultures of the bacteriocin producing strain, to determine the standard deviation and confirm antimicrobial action.

***Tips:***

1. All media should be prepared on the day of use to ensure it is of the best quality.
2. Keep the volumes of agar in a water bath or incubator right up until the time they are required, as agar solidifies quickly and can form clumps when poured.
3. Before punching holes in the agar, allow the rod to cool within range of the Bunsen flame to maintain sterility. Doing so makes it easier to remove the agar plugs, as the heated rod can liquify them.
4. When removing the agar plugs with a pipette tip, skewer the plugs at an angle and lift out to avoid making tears in the surrounding media.


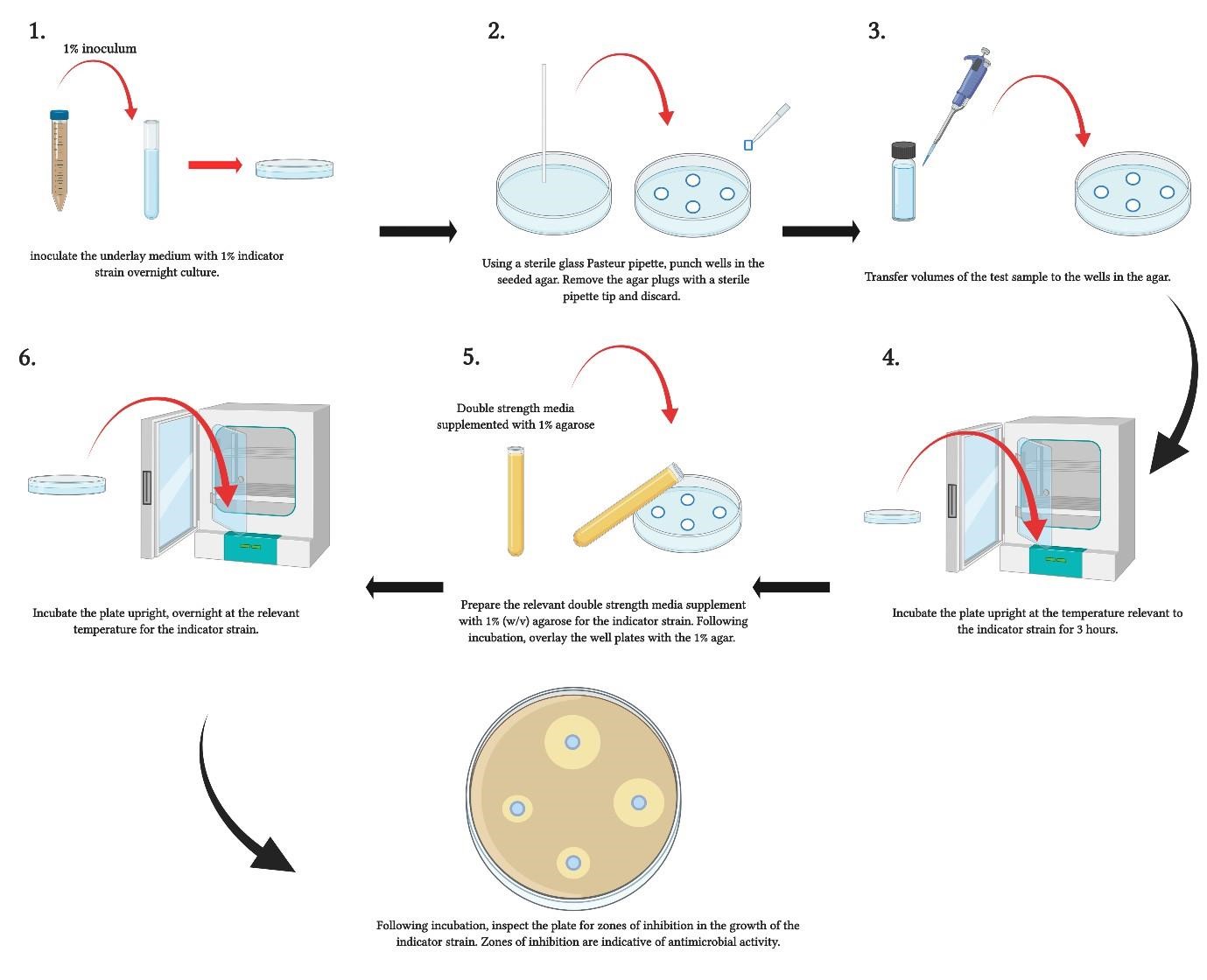


**Figure S4: Agarose-based radial diffusion assay to detect the presence of antimicrobial activity. Zones of inhibition in the overlay layer indicate antimicrobial action. This test can be used to detect bioactivity in samples containing low concentrations of the antimicrobial under investigation. It can also be used to detect activity against Gram-negative organisms. Created using Biorender.com.**

**Protocol S6: Purification of bacteriocin peptides**

1. Prepare a culture of the bacteriocin producing strain, as per **Protocol S1** at a final volume of 20mL.
2. Following incubation, prepare 1980mL of fresh, relevant broth. Inoculate the fresh broth with the 20mL overnight culture to create a 1% inoculum in a final volume of 2L.
3. Incubate the 2L culture for 20 hours at the relevant temperature
4. Following incubation, centrifuge 2L overnight culture at 7000 x *g* for 20 minutes, (Sorvall RC 6+ model centrifuge, Thermo Scientific, product code. 12121680).
5. Prepare the cell free supernatant as previously described in **Protocol S3**, steps 2 – 3. Aseptically transfer the cell free supernatant to sterile flasks.
6. Load a 30cm long, glass chromatography column with an internal diameter of 2.5cm, (Merk, product no. C4419-1EA), with 60g of Amberlite XAD-16N beads (Sigma Aldrich, product no. XAD16-500G).
7. Wash the beads with 1L of sterile distilled water before passing the CFS (from step 5) through the column.
8. When the CFS has completely passed through, wash the beads with 500mL of 30% ethanol (Sigma Aldrich, CAS no: 64-17-5).
9. Once the beads have been washed with ethanol, elute the bound peptide using 500mL of 70% isopropanol 0.1% trifluoroacetic acid. Retain the eluted material.
10. Resuspend the cell pellet from the overnight culture (obtained in step 5) in 250mL of 70% isopropanol 0.1% TFA.
11. Transfer the resuspension to a flask.
12. Using a magnetic stirring bar, stir for 3 hours at room temperature.
13. Following the three-hour incubation period, centrifuge at 7000 x *g* for 20 minutes. Retain the supernatant, (now referred to as the whole cell extract), and discard any cellular debris.
14. Combine the eluate from step 9, with the whole cell extract from step 13, as peptide is being purified from both elements.
15. Place combined components into a rotary evaporator, (Rotavapor R-300, Buchi), and concentrate to remove isopropanol. Isopropanol is considered removed when the solution has been reduced to volume of 250mL.
16. Adjust the pH of the solution to pH 4 before applying to a 10g (60 ml), SPE C-18 Bond Elute Column (Phenomenex), pre-equilibrated with 60ml methanol (Sigma Aldrich, product no. 34860-1L-R) and 60ml water.
17. Wash the solution through the column using 120mL 30% ethanol.
18. Elute bound peptide using a 60mL solution of 70% isopropanol, 0.1% trifluoroacetic acid and distilled water.
19. Divide the 60mL solution into four 15mL volumes, then concentrate each 15mL volume to 2mL volumes through further rotary evaporation to remove any remaining isopropanol prior to HPLC.

# Peptide purification through RP HPLC

1. Run each 2mL volume of the concentrated solution through a Phenomenex (Phenomenex, Cheshire, UK), C12 reverse phase HPLC column (Jupiter 4u proteo 90 Å, 250×10.0 mm, 4 µm), equilibrated with 25% acetonitrile and 0.1% trifluoroacetic acid. A gradient of 30– 50% acetonitrile (Fisher, CAS no. 75-05-8) containing 0.1% trifluoroacetic acid facilitates the separation of each fraction.
2. Adjust the flow rate of the sample to 3.2mL per minute.
3. When the HPLC run is complete, retain all fractions.

**Determining the relevant HPLC fraction.**

1. Perform a well diffusion assay as previously described in **Protocol S4**, steps 2 – 8.
2. Transfer 30µL volumes of each retained fraction to individual wells of agar seeded with a relevant indicator strain, (1% inoculum).
3. Store the HPLC fractions at 4^o^C overnight while incubating the well diffusion plates upright overnight at the relevant temperature for the indicator strain.
4. Following incubation, inspect the plates for zones of clearing surround the wells. Note the wells and the corresponding fraction that formed the clearings in the growth of the indicator strain, as these are the fractions containing the active peptide.

Perform well assays in triplicate for each fraction to ensure that the antimicrobial activity is appropriately assessed.

# Preparing freeze dried peptide

1. Pool all fractions showing bioactivity and remove any remaining acetonitrile through further rotary evaporation. Any fractions which do not display activity can be discarded.
2. Purified fractions can then be freeze dried, (FreezeZone 6 bench top freeze dryer, Labconco, cat no. 7752020) and stored at -20^o^C.

# Purity of the purified peptide

29. The purified peptide should be subjected to MALDI-ToF Mass Spectrometric analysis to confirm purity before use.

***Tips:***

1. Once centrifugation is complete, transfer the supernatant as quickly as possible to prevent the pellet becoming resuspended in the supernatant.
2. Once centrifugation is complete, transfer the whole cell extract to a conical flask (Erlenmeyer flask) as quickly as possible to prevent the cell debris becoming resuspended in the whole cell extract supernatant.
3. MS analysis of the purified product must be carried out to ensure the product is pure.

**Protocol S7(a): Stability assay - incubation with protease enzymes**

**See *Figure S5* for protocol diagram.**

**Preparing indicator strain**

1. Prepare a culture of the indicator strain as per **Protocol S1**, at a final volume of 10mL.

# Protease exposure

1. Resuspend lyophilised protease enzyme at a concentration in a solution of 100 mM TrisHCl, (Tris, Sigma Aldrich, cat no. 10708976001; HCl, Thermo Fisher, product no. 10467640) and 10 mM CaCl_2_ (Sigma Aldrich, cat no. 449709-10G), at a final concentration of 100mg/mL.
2. Transfer 800µL test sample and 200µL of resuspended enzyme to a sterile Eppendorf tube, to the enzyme is at a final concentration of 20mg/mL.
3. Vortex the Eppendorf and incubate at 37^o^C for four hours.

# Determining protease stability

1. Perform a well diffusion assay as previously described in **Protocol S4**, steps 2 -8.
2. Aseptically transfer 30µL volumes of the enzyme exposed test sample and the control, (test sample in the absence of enzyme), to individual wells in the agar.
3. Incubate plates upright at the relevant temperature for the indicator strain overnight.
4. Inspect the zones of inhibition surrounding the wells. A reduction in zone size for the supernatant exposed to protease enzyme indicates the protease susceptibility and the presence of an antimicrobial peptide.

Perform in triplicate using three biological repeats, i.e. three separate overnight cultures of the bacteriocin producing strain, to determine the standard deviation and confirm antimicrobial action.

***Tips:***

1. All media, resuspended peptides, cell free supernatant samples and resuspended enzymes should be prepared on the day of use to ensure it is of the best quality.
2. This test can be performed used the resuspended purified peptide or the cell free supernatant of the isolated culture.
3. We suggest testing antimicrobial sensitivity against a range of proteases, e.g. proteinase K (Sigma Aldrich, cat no. P2308-100MG), α-chymotrypsin (Sigma Aldrich, cat no. T1426-100MG) and pepsin (Sigma Aldrich, cat. No P7000-100G), to determine the complete range of susceptibility.


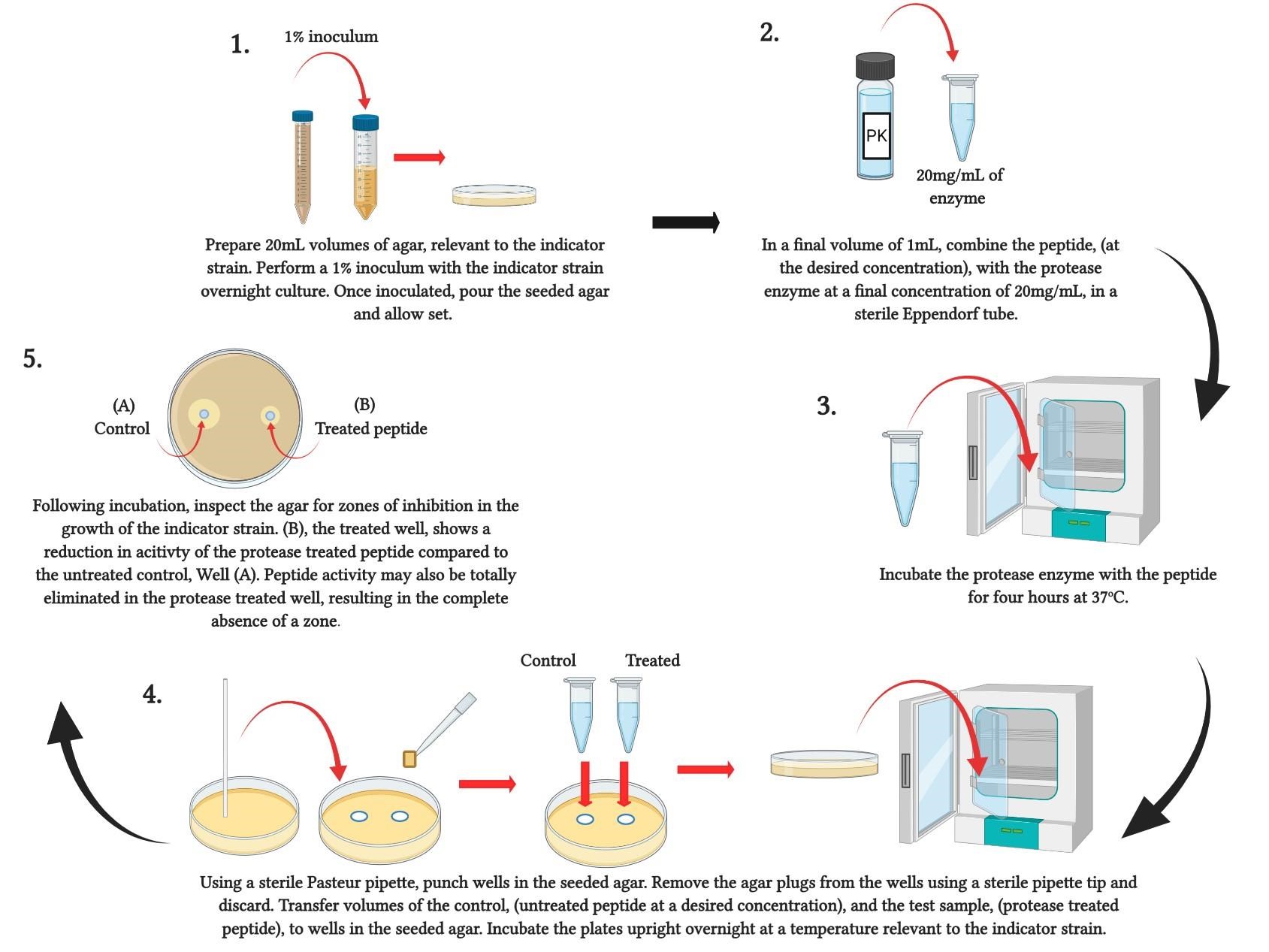


**Figure S5: Well diffusion assay to determine protease susceptibility of antimicrobial under investigation. If the antimicrobial is sensitive to proteases, incubation with the enzyme will cause the zone of clearing in the indicator strain growth to be reduced. Created using Biorender.com.**

**Protocol S7(b): Susceptibility assays - crescent moon protease assay**

**See *Figure S6* for protocol diagram.**

# Preparing cultures for proteinase testing

1. Prepare a culture of the isolated strain under investigation, as per **Protocol S1** at a final volume of 10mL.
2. Spot 20uL of the overnight culture on to the surface of a fresh agar plate. Allow spotted supernatant to dry a sterile biological safety cabinet.
3. Once dry, invert plates and incubate for 16 – 18 hours at the relevant temperature for the isolate.

# Preparing indicator strain

1. Prepare a culture of the indicator strain as per **Protocol S1**, at a final volume of 10mL.

**Determining protease susceptibility.**

1. Following incubation, remove the lids of the spot plates within the UV box, and expose the spotted cultures, (cultures facing the bulbs), to the maximum wavelength for 30 minutes.
2. Resuspend lyophilised protease enzyme in a solution of 100 mM Tris-HCl, (Tris, Sigma Aldrich, cat no. 10708976001; HCl, Thermo Fisher, product no. 10467640) and 10 mM CaCl_2_ (Sigma Aldrich, cat no. 449709-10G), at a final concentration of 20mg/mL.
3. Spot 20µL of the resuspended protease next to the UV treated culture on the surface of the plate, then incubate the UV treated culture and the proteinase for 1 hour at 37^o^C.
4. Prepare 0.75% w/v “sloppy” agar, divide into 10mL volumes, and bring to a temperature of 50ºC using an incubator or water bath.
5. Inoculate each 10mL volume with 100µL of indicator strain overnight culture, (1% inoculum).
6. Following incubation, overlay the spot culture with the inoculated 0.75% w/v “sloppy” agar.
7. Invert the plates and incubate overnight at the relevant temperate for the indicator strain. Inspect the zones of inhibition in the overlaid media. The presence of an indentation, giving a crescent like appearance to the zone of inhibition, indicates protease susceptibility and the presence of an antimicrobial peptide.

Perform in triplicate using three biological repeats, i.e. three separate overnight cultures of the bacteriocin producing strain, to determine the standard deviation and confirm antimicrobial action.

***Tips:***

1. All media, resuspended peptides, cell free supernatant samples and resuspended enzymes should be prepared on the day of use to ensure it is of the best quality.
2. This test can be performed using the resuspended purified peptide or the cell free supernatant of the isolated culture.
3. We suggest testing antimicrobial sensitivity against a range of proteases, e.g. proteinase K (Sigma Aldrich, cat no. P2308-100MG), α-chymotrypsin (Sigma Aldrich, cat no. T1426-100MG) and pepsin (Sigma Aldrich, cat. No P7000-100G), to determine the complete range of susceptibility.
4. If spotting the protease enzyme next to the cell free supernatant on the surface of the agar, allow spots dry in a sterile biological safety cabinet prior to incubation in order to prevent the spot from spreading or moving on the plates.


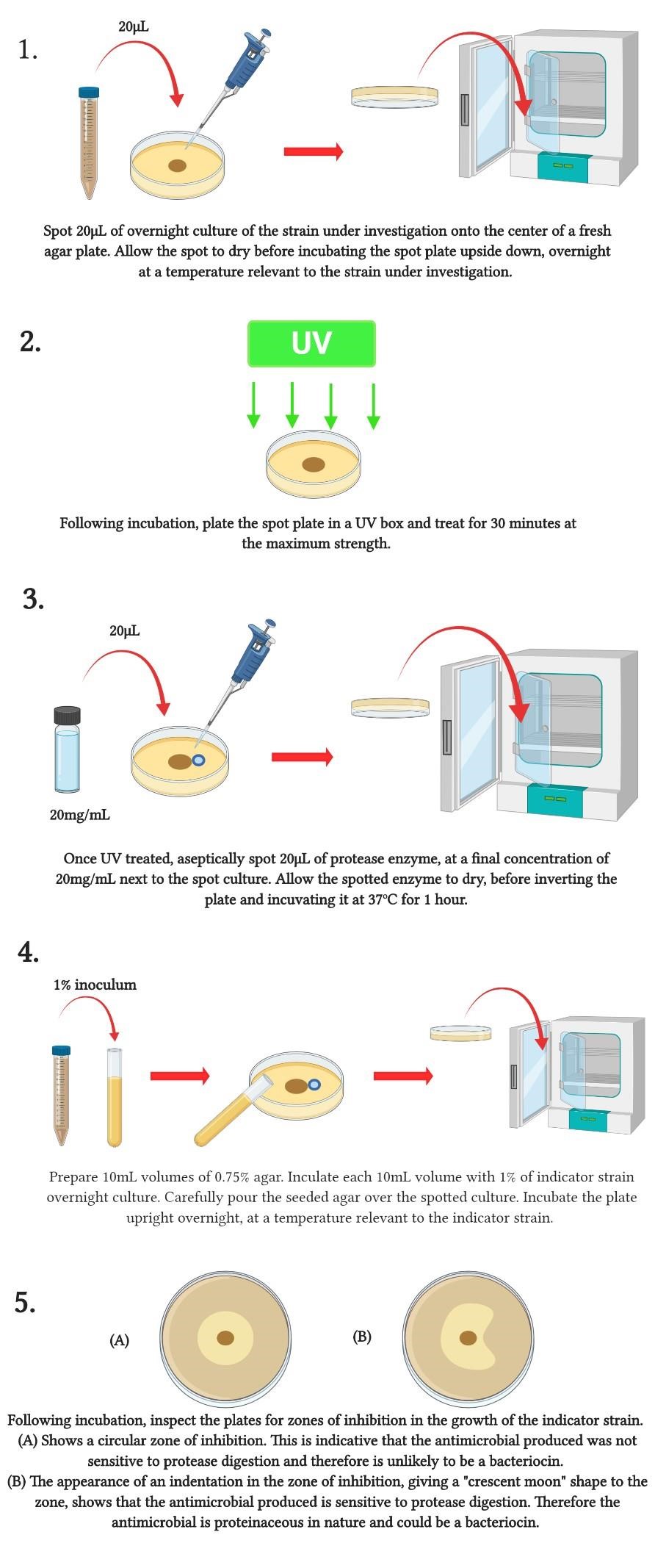


**Figure S6: Protease spot test to determine antimicrobial sensitivity to proteolytic enzymes. If the antimicrobial under investigation is sensitive to the protease tested, the zone of clearing will be impacted. A crescent moon shaped zone, rather than a round zone of clearing, will appear in the indicator strain growth, as the antimicrobial in contact with the enzyme will be inactivated. Created using Biorender.com.**

**Protocol S7(c): Susceptibility assay – pH treatment**

# Preparing cultures

1. Prepare a culture of the isolated strain under investigation, as per **Protocol S1** at a final volume of 10mL.
2. Prepare a culture of the indicator strain as per **Protocol S1**, at a final volume of 10mL.

**Determining pH sensitivity.**

1. Following incubation, obtain the CFS of the strain under investigation as per **Protocol S3**, steps 2 – 4.
2. Aseptically determine the pH of the CFS using a calibrated pH meter, (Sigma Aldrich, Product no. Z655295).
3. Using 1M HCl (Sigma Aldrich, Product no. H1758) and 1M NaOH (Sigma Aldrich, Product no. S8045), adjust the pH of the CFS until the desired pH is achieved, (see **Tip (a)** for the suggested pH).
4. When the desired pH has been achieved, incubate the pH treated samples for 4 hours at room temperature.
5. Following incubation, perform a well diffusion assay as per **Protocol S4**, steps 2 – 8, where the agar in inoculated with the indicator strain, the pH adjusted CFS is the test sample and using the unadjusted CFS as a control.
6. Incubate the plates upright overnight at a temperature relevant to the indicator strain.

Following incubation examine the agar for zones of clearing around the wells.

Perform in triplicate using three biological repeats, i.e. three separate overnight cultures of the bacteriocin producing strain, to determine the standard deviation and confirm antimicrobial action.

***Tips:***

1. When assessing the pH sensitivity of an antimicrobial, we recommend a broad range of pHs, (from pH 2 – pH 11), be investigated in order to determine the full spectrum of stability.
2. The cell free supernatant should be used on the day they are prepared. This ensures the solutions are fresh and that no bioactivity has been lost.
3. Keep the volumes agar in a water bath or incubator right up until the time they are required, as agar sets quickly and can form clumps when poured.
4. Once centrifugation is complete, transfer the supernatant as quickly as possible to a separate sterile container from the pellet or cellular debris. This prevents the pellet from becoming resuspended in the supernatant.
5. Supernatants can be filter sterilised using a 0.45µm pore filter, (Sarstedt, order no.

83.1826), to ensure that all cells have been removed.

1. Gently vortex the supernatant before use.

**Protocol S7(d): Susceptibility assay – temperature treatment**

# Preparing cultures

1. Prepare a culture of the isolated strain under investigation, as per **Protocol S1** at a final volume of 10mL.
2. Prepare a culture of the indicator strain as per **Protocol S1**, at a final volume of 10mL.

# Determining temperature stability

1. Following incubation, obtain the CFS of the strain under investigation as per **Protocol S3**, steps 2 – 4. Ensure that the CFS has been transferred to a suitable container that can withstand the potentially high temperature the sample will be subjected to (Fisher Scientific Product Code. 13094697).
2. Set a calibrated heat block (Fisher Scientific, cat. no. 88-870-007) to the desired temperature. Ensure the heat block has reached the desired temperature before placing the sample in the heat block. Place the tubes containing the CFS samples into the heat block and incubate at the desired temperature for three hours.
3. Following incubation, perform a well diffusion assay as per **Protocol S4**, steps 2 – 4, inoculating the agar with the indicator strain, using the heat treated CFS as the test sample and untreated CFS as a control.
4. Incubate the plates upright overnight at a temperature relevant to the indicator strain.

Following incubation examine the agar for zones of clearing around the wells.

Perform in triplicate using three biological repeats, i.e. three separate overnight cultures of the bacteriocin producing strain, to determine the standard deviation and confirm antimicrobial action.

***Tips:***

1. When assessing how temperature effects antimicrobial activity, we recommend the sample be subjected to a broad range of temperatures, (0^o^C, 10^o^C, 20^o^C, 25^o^C, 30^o^C, 37^o^C, 40^o^C, 50^o^C, 65^o^C, 80^o^C, 90^o^C, 100^o^C, 121^o^C), in order to determine the full spectrum of stability.
2. The cell free supernatant should be used on the day they are prepared. This ensures the solutions are fresh and that no bioactivity has been lost.
3. Keep the volumes agar in a water bath or incubator right up until the time they are required, as agar sets quickly and can form clumps when poured.
4. Once centrifugation is complete, transfer the supernatant as quickly as possible to a separate sterile container from the pellet or cellular debris. This prevents the pellet or debris from becoming resuspended in the supernatant.
5. Supernatants can be filter sterilised using a 0.45µm pore filter, (Sarstedt, order no.

83.1826), to ensure that all cells and debris have been removed.

1. Gently vortex the supernatant and whole cell extract before use.

**Protocol S8: Minimum inhibitory concentration assay**

**See *Figure S7* for protocol diagram.**

**Preparation of the of test bacterium culture.**

1. Prepare a culture of the test bacterium as per **Protocol S1**, in a final volume of 10mL.
2. Following incubation, subculture the overnight culture of test bacterium into 10mL of fresh broth and incubate at the relevant temperature for the strain, until an OD600nm of 0.5 is obtained.
3. Standardise the cells by inoculating 980µL of fresh broth with 20µL of culture, (**see Tip (a) for notes on the standardisation of cells**).

**Preparing the microtiter plate.**

1. Add 100µL of relevant, sterile broth to each well in a row of a treated, (**see Tip (b)** for microtiter plate treatment), 96 well plate, (Round well, flat bottomed microtiter plate with lid; Sarstedt, order no. 82.1582.001).
2. Resuspend the purified peptide in the relevant broth, then transfer 100µL of resuspended peptide to the first well containing broth, to create a twofold dilution.
3. Perform a twofold dilution of the peptide along the row of wells containing broth, (transfer 100µL from the well containing peptide to the next well containing only broth).

Each well should contain a final volume of 100µL of peptide in the relevant broth.

# Calculating the MIC

1. Dilute the standardised cells from **Step 3**, by inoculating 100µL into 10mL of fresh broth.
2. Aseptically transfer 100µL of the diluted culture to each well of the microtiter plate then incubate the plate at the relevant temperature for the test bacterium for 16 hours.
3. Following incubation, inspect the wells for turbidity or growth. The MIC of the peptide is the lowest concentration completely inhibits visible growth as judged by the naked eye.

Perform in triplicate using three biological repeats, i.e. three separate overnight cultures of the test bacterium, to determine the standard deviation and confirm antimicrobial action.

***Tips:***

1. When incubating the biological repeats of the subculture, it should be noted that they may not all achieve an OD600nm of 0.5 simultaneously. To achieve the same concentration of cells in all repeats, standardisation is performed. If an OD600nm of 0.4 is reached, the subculture can be standardised by inoculating 25µL into 975µL of fresh broth, as this will achieve the same CFU/mL concentration as a subculture of 20µL OD600nm of 0.5 culture into 980µL.
2. Prior to use, the plastic microtiter place should be treated to reduce unfavourable interactions between the peptide and the plate surface. Dissolve bovine serum albumin (Sigma Aldrich, CAS no. A9418-50G) at a concentration of 1% (wt/vol) in 10mM phosphate buffer solution (Sigma Aldrich, product no. P5244-100ML). Filter sterilise this solution with a 0.45µm filter (Sarstedt, order no. 83.1826). Using a multichannel pipette, (Eppendorf, 12-channel variable pipette, cat no. 3125000028), transfer 200µL of this solution each well of a 96 well microtiter plate (Round well, flat bottomed microtiter plate with lid; Sarstedt, order no. 82.1582.001) and incubate the plate for 30 mins at 37ºC. Following incubation, use the multichannel pipette to remove the solution from the wells. Rinse each well with 200µL of sterile 10mM phosphate buffer solution and remove. Allow the plate to dry in sterile biological safety cabinet.
3. When transferring volumes between wells, ensure the pipette tip is changed each time to prevent cross-contamination.


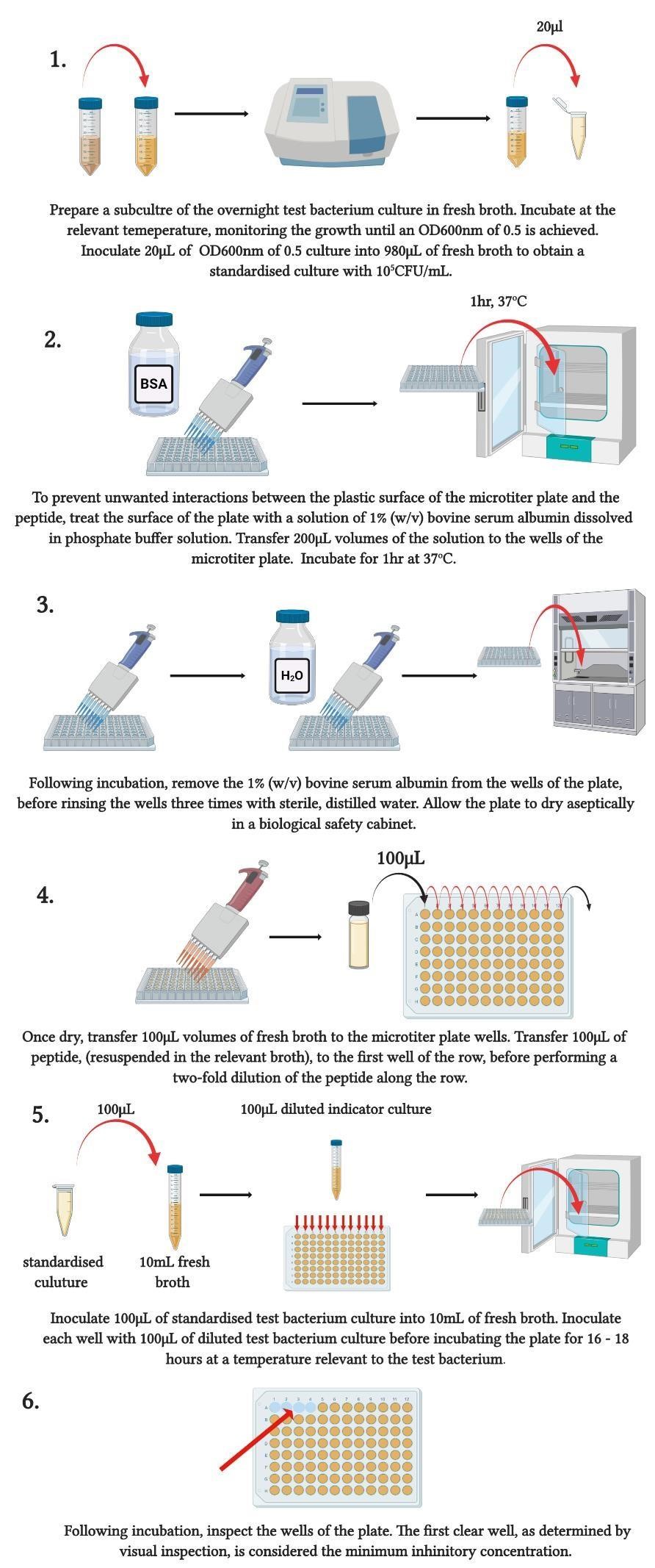


**Figure S7: Broth microdilution method to detect the minimum inhibitory concentration of an antimicrobial. The minimum inhibitory concentration is defined by the European Committee on Antimicrobial Susceptibility Testing as “the lowest concentration of the agent that completely inhibits visible growth as judged by the naked eye, disregarding a single colony or a thin haze within the area of the inoculated spot”. Created using Biorender.com.**

**Protocol S9: Growth assay.**

**See *Figure S8* for protocol diagram.**

**Preparation of test bacterial culture.**

1. Prepare a culture of the test bacterium as per **Protocol S1**, in a final volume of 10mL.

# Growth assay

1. Resuspend the peptide in sterile.
2. Transfer 100µL of the overnight test bacterium culture to individual wells of a sterile 96 well plate (Round well, flat bottomed microtiter plate with lid; Sarstedt, order no. 82.1582.001).
3. Add 100µL volumes of resuspended peptide to the wells containing overnight culture, where the peptide is at a known concentration, (see *Tip (a)* for peptide concentrations), in a final volume of 200µL.
4. To create a control, (growth of the bacterium in the absence of an antimicrobial), add 100µL of the overnight test bacterium culture to individual wells of a sterile 96 well plate. Next, add 100µL of sterile broth to the wells containing the bacterial culture.
5. Monitor cell growth spectrophotometrically at 600nm over a 24-hour period, taking readings every 60 minutes, using an automated spectrophotometric plate reader, (Varioskan LUX multimode microplate reader, Thermo Fisher, cat no. VL0000D0), at a temperature relevant to the test bacterium.

Perform in triplicate using three biological repeats, i.e. three separate overnight cultures of the test bacterium, to determine the standard deviation and confirm antimicrobial action.

***Tips:***

a. Resuspend the peptide at a variety of concentrations, both above and below the MIC, and expose the indicator strain to each concentration variation.. This will allow a better assessment of bacteriocin effects on bacterial growth.


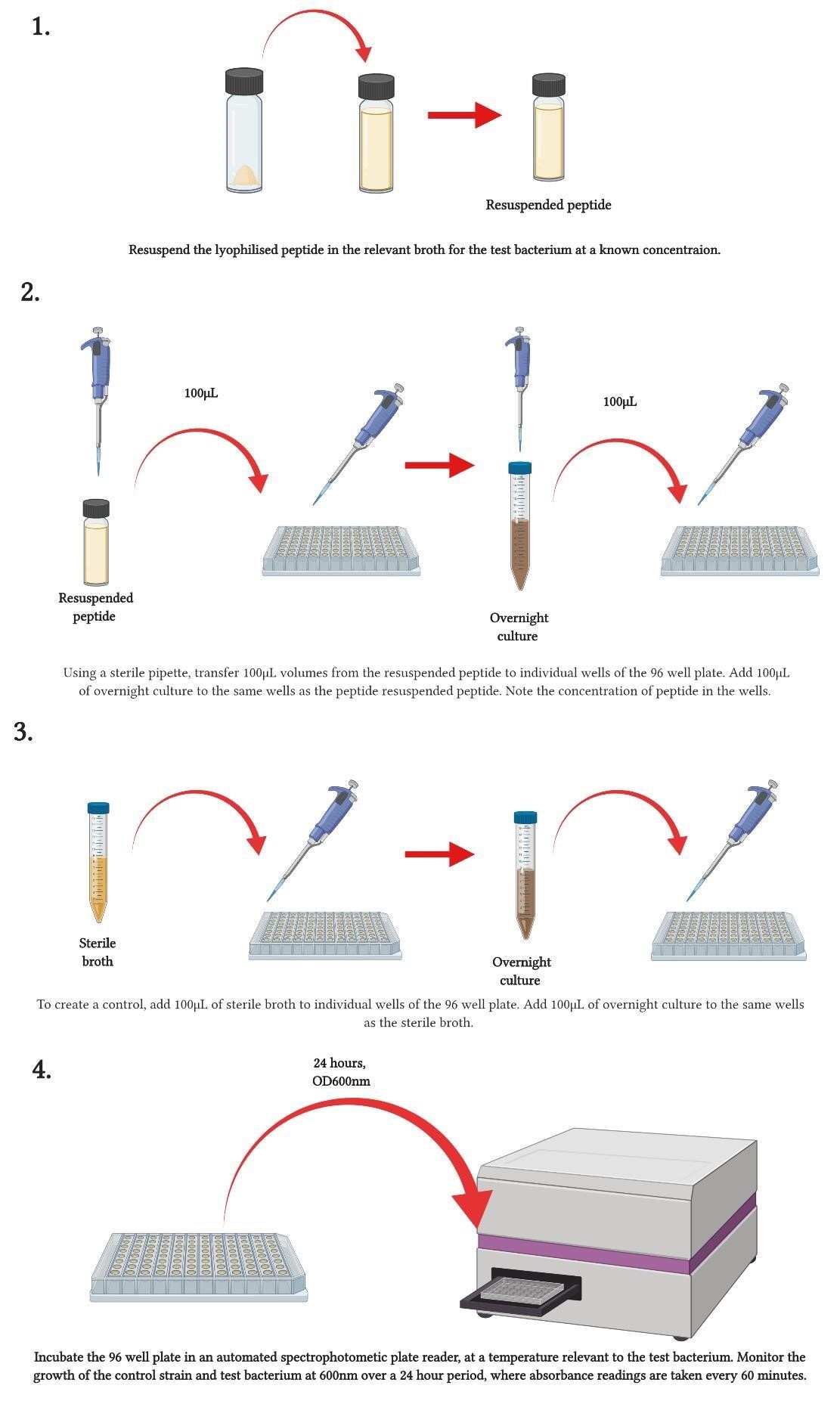


**Figure S8: Growth curve assay to assess the impact the peptide has on bacterial culture growth. Minimum inhibitory concentrations are considered “end point assays” meaning that a singular result is obtained once the test is complete and the reaction has been stopped. Kinetic assays, like growth curves, provide insight into the effects of an antimicrobial on cell growth and death over the duration of the test at multiple time points. Created using Biorender.com.**

**Protocol S10: Kill curve assay.**

**See *Figure S9* for protocol diagram.**

**Preparation of test bacterium culture.**

1. Prepare a culture of the test bacterium as per **Protocol S1**, in a final volume of 10mL.

# Kill curve assay

1. Resuspend the lyophilised peptide in the relevant broth.
2. Combine the test bacterium overnight culture with the resuspended peptide to achieve in a final volume of 1mL, where the test bacterium has a final concentration of 10^7^CFU/ml and peptide is at a known, desired concentration (see *Tip (a)* for peptide concentrations), in a sterile Eppendorf tube.
3. Before incubation, remove 100µL from the peptide – test bacterium culture and perform a serial dilution using sterile ¼ strength Ringers solution until a 10^-6^ dilution is obtained (Sigma Aldrich, product number 96724-100TAB).
4. Plate 100µL volumes of each dilution on individual agar plates of the relevant media type. Ensure the dilutions are spread evenly over the surface of the plate using a sterile plate spreader, (Merk, product no. HS8171). This is considered the T0 cell count.
5. Vortex the Eppendorf and incubate the remaining volume at the relevant temperature.
6. At chosen time points, remove 100µL volumes from the Eppendorf tube. Repeat steps

(4) – (6), clearly noting the time points at which each plated dilution was taken.

1. When all time points have been collected, invert the dilution plates and incubate them overnight at the relevant temperature for the test bacterium.
2. Perform colony counts on each plate and calculate the CFU/mL for each time point.

Perform in triplicate using three biological repeats, i.e. three separate overnight cultures of the test bacterium, to determine the standard deviation and confirm antimicrobial action.

***Tips:***

a. Resuspend the peptide at a variety of concentrations, both above and below the MIC, and expose the test bacterium to each. This will allow a better assessment of bacteriocin effects on bacterial growth.


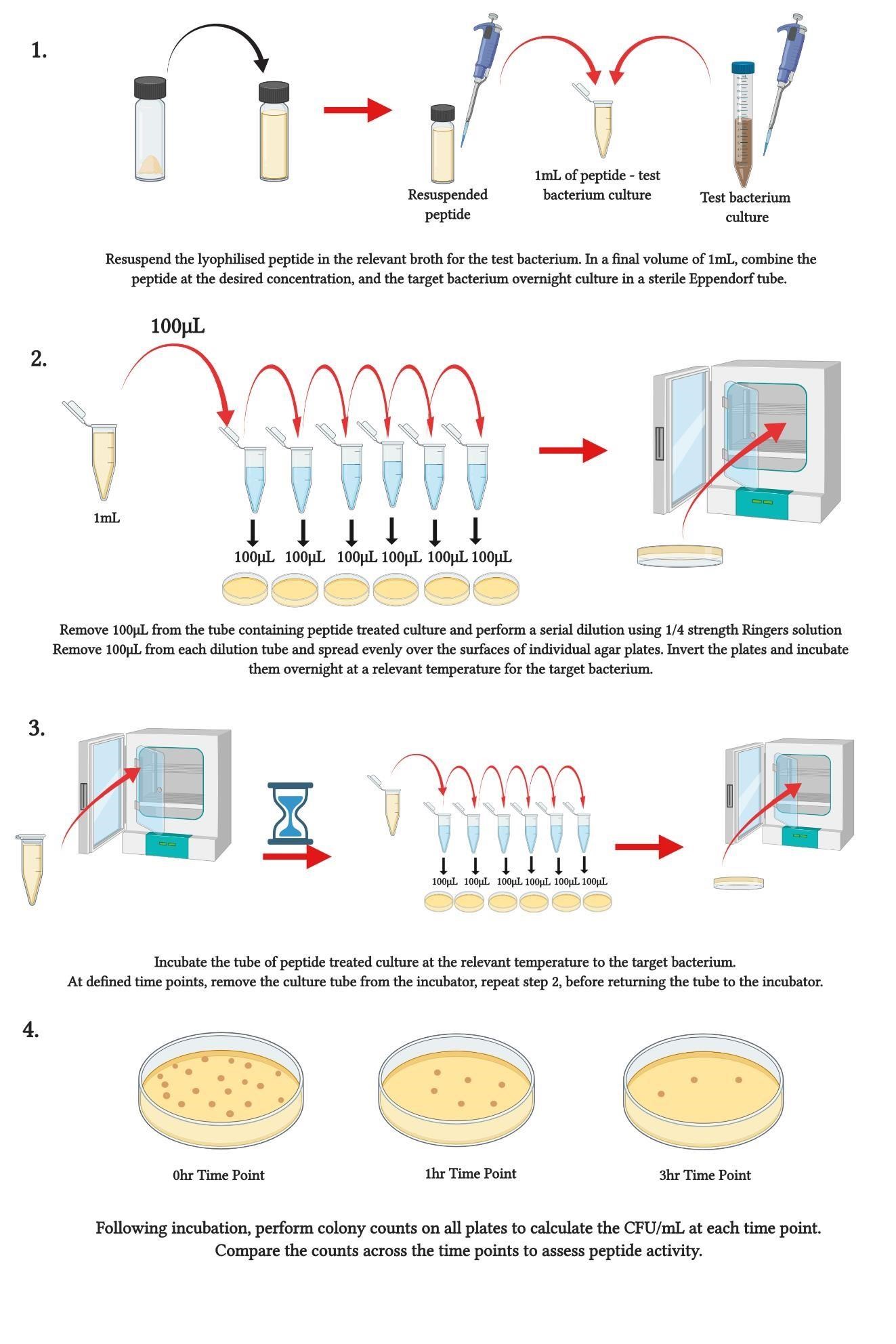


**Figure S9: Kill curve assay. By taking samples of the peptide exposed culture at different time points, the kill effects of the bacteriocin can be assessed. Created using Biorender.com.**

**Protocol S11(a): Model food trials with a liquid food sample**

# Selection of a food sample

1. Purchase a relevant liquid, commercial food, (see **Tip (a)** for the selection of an appropriate food sample), and plate 100µL of said sample on agar that is selective for the test bacterium that will be used during the study, e.g. Listeria Select Agar or Chromogenic Cronobacter Isolation agar. Incubate the plate overnight at a temperature relevant to the test bacterium.
2. Following incubation inspect the plates for growth. If the food is found to be free of the test bacterium, it is suitable if use in the model food trial. If intrinsic contamination is detected, the sample should be discarded.

# Preparing the test bacterium culture

1. Prepare a culture of the test bacterium as per **Protocol S1**, in a final volume of 10mL.

**Model food trial.**

1. Aseptically transfer the desired volume of the liquid food to a sterile 1.5mL Eppendorf tube.
2. Aseptically add the lyophilised peptide to the liquid test sample at a desired concentration, (see **Tip (a)** for suggested peptide concentrations).
3. Inoculate the sample with the test bacterium concentration at a controlled concentration, (see **Tip (b)** for recommended concentrations of the test bacterium).
4. Incubate the inoculated food sample at a temperature relevant to the real-life storage conditions of the food sample, (see **Tip (c)** for incubation temperature recommendations).
5. Periodically over the course of the incubation, (see **Tip (d)** for suggested incubation time points), aseptically remove 1mL of the sample and perform a serial dilution using sterile ¼ strength Ringer solution (Sigma Aldrich, product number 96724-100TAB).
6. Using a sterile spreader (Merk, product no. HS8171), spread 1mL of each dilution over the surface of an agar plate selective for the test bacterium, before incubating the plate overnight at a temperature relevant to the test bacterium.
7. Following incubation, perform a CFU/mL count on the spread plates.

Perform in triplicate using three biological repeats, i.e. three separate overnight cultures of the test bacterium, to determine the standard deviation and confirm antimicrobial action.

***Tips:***

- 1. Resuspend the peptide at a variety of concentrations, both above and below the MIC, and expose the test bacterium to each concentration variation. This will allow a better assessment of bacteriocin effects on bacterial growth in a complex medium.
  2. Under real life conditions, contaminating organisms are typically present in very low concentrations within food, (e.g. *L. monocytogenes* has been found at 10^2^ CFU/mL (Spanu et al., 2014)). This concentration of cells would however be too low to detect through conventional plating methods. For this reason, a higher starting inoculum is suggested so the CFU/mL can be determined. We suggest inoculating at the food samples at 10^5^CFU/mL. This concentration is high enough to allow detection through plating, while remaining as close to a “real” initial inoculum as possible.
  3. When incubating the model food sample with the peptide, select a temperature that is relevant to the typical storage conditions of the food, (refrigerated at 4^o^C or between 20^o^C and 25^o^C to reflect room temperature). Once typical storage conditions have been investigated, the test should be repeated at a range of temperatures to assess the durability of the peptide and the impact of temperature on bioactivity in a complex medium, e.g. at stressful storage conditions (excessively high or low temperatures).
  4. Unlike a kill or growth curve, where samples are monitored over the period of hours or a day, a model food trial should be tested over a more extended period to best reflect food storage conditions. We recommend taking samples daily for a period of 11 days, and then, weekly up to 7 weeks, (Kara et al., 2014; Vijayakumar and Muriana, 2017).
  5. The pH of the food should be tested and composition of food, e.g. percentage fats and sugars, should be noted. It is important to record the conditions and environment the bacteriocin is exposed to, in order to note which complex media types it may and may not be effective in.

**Protoco1 S11(b): Model food trials with a solid food sample**

# Selection of a food sample

1. Purchase a relevant solid commercial food sample, (see **Tip (a)** for the selection of an appropriate food sample). Take a section or piece of the food sample and aseptically transfer to a volume of sterile quarter strength Ringer solution (Sigma Aldrich, product number 96724-100TAB) and incubate at room temperature for 10 minutes. Following incubation aseptically spread 100µL of the Ringer solution on the surface of an agar plate which is selective for the test bacterium e.g. Listeria Selective Agar. Incubate the plate overnight at a temperature relevant to the test bacterium.
2. Following incubation inspect the plates for growth. If the food sample is found to be free of the test bacterium, it is suitable if use in the model food trial. If intrinsic contamination is detected, the sample should be discarded.

# Preparing the test bacterium culture

1. Prepare a culture of the test bacterium as per **Protocol S1**, in a final volume of 10mL, (see **Tip (a)** for the selection of an appropriate test bacterium).
2. Aseptically divide the food sample into suitable quantities that will last the duration of the sampling, e.g. 5g portions.
3. Aseptically dip each weighed test sample into a solution of peptide resuspended in sterile, distilled water at a desired concentration (see **Tip (b)** for suggested peptide concentrations).
4. Allow test samples to dry aseptically in a biological safety cabinet before placing in a sterile container.
5. Aseptically inoculate the food sample with 1mL of the test bacterium at a controlled concentration, (see **Tip (c)** for recommended test bacterium concentrations).
6. Incubate the inoculated food sample at a temperature relevant to the real-life storage conditions of the food sample, (see **Tip (d)** for incubation temperature recommendations).
7. Periodically over the course of the incubation, (see **Tip (e)** for suggested incubation time points), aseptically remove a predetermined quantity of the food sample from the container. Transfer the obtained sample to a sterile bag with sterile Ringer solution, (1:10 dilution), before homogenising with a stomacher or macerating by hand.
8. Using a sterile spreader (Merk, product no. HS8171), spread 100µL of this homogenised solution over the surface of an agar plate selective for the test bacterium, before incubating the plate overnight at a temperature relevant to the test bacterium.
9. Following incubation, perform a CFU/mL count on the spread plates.

Perform in triplicate using three biological repeats, i.e. three separate overnight cultures of the test bacterium, to determine the standard deviation and confirm antimicrobial action.

***Tips:***

1. When designing a model food trial, it is important to consider the following:

- When selecting a food type to sample, we suggest selecting one that has previously been linked with contamination of solid foods and food borne illness in order to make the model food trial as realistic and reflective of market conditions as possible, e.g. chocolate milk (Hanson et al., 2019), apple juice (Cody et al., 1999) or infant formula (FAO and WHO, 2006).

1. Under real life conditions, contaminating organisms are typically present in very low concentrations within food, (e.g. *L. monocytogenes* has been found at 10^2^CFU/mL (Spanu et al., 2014)). This concentration of cells would however be too low to detect through conventional plating methods. For this reason, a higher starting inoculum is suggested so the CFU/mL can be determined. We suggest inoculating at the food samples at 10^5^CFU/mL. This concentration is high enough to allow detection through plating, while remaining as close to a “real” initial inoculum as possible.
2. When incubating the model food sample with the peptide, select a temperature that is relevant to the typical storage conditions of the food, (refrigerated at 4^o^C or 20^o^C to reflect room temperature). Once typical storage conditions have been investigated, the test should be repeated at a range of temperatures to assess the durability of the peptide and the impact of temperature on bioactivity in a complex medium, e.g. at stressful storage conditions (excessively high or low temperatures).
3. Unlike a kill or growth curve, where samples are monitored over the period of hours or a day, a model food trial should be tested over a more extended period to best reflect food storage conditions. We recommend taking samples daily for a period of 11 days, and then, weekly up to 7 weeks (Kara et al., 2014; Vijayakumar and Muriana, 2017).
4. The pH of the food should be tested and composition of food, e.g. percentage fats and sugars, should be noted. It is important to record the conditions and environment the bacteriocin is exposed to, in order to note which complex media types it may and may not be effective in.

**Protocol S12: Inhibition of biofilm on plastic surfaces.**

**See *Figure S10* for protocol diagram.**

# Preparing the test bacterium culture

1. Prepare a culture of the test bacterium as per **Protocol S1**, supplementing the medium with 1% (w/v) D-(+) glucose, (see *Tip (a)*), in a final volume of 10mL.

# Biofilm inhibition assay

1. Resuspend the lyophilised peptide in the relevant broth supplemented with 1% (w/v) D(+) glucose (Sigma Aldrich, product no. G8270-1KG).
2. Perform a 1:100 dilution on the test bacterium overnight culture by inoculating 10µL of culture into 990µL of relevant broth, supplemented with 1% (w/v) D-(+) glucose and the peptide at the desired concentration, (see *Tip (b)* for peptide concentration), in a final volume of 1mL.
3. Create a control by performing a 1:100 dilution on the test bacterium overnight culture, (10µL of culture inoculated into 990µL of relevant broth, supplemented with 1% (w/v) D-(+) glucose), in the absence of the peptide.
4. Transfer 200µL volumes of the peptide- test bacterium culture and the control culture to individual wells of a sterile 96 well microtiter plate (Round well, flat bottomed microtiter plate, with a lid; Sarstedt, order no. 82.1582.001).
5. Incubate at the relevant temperature for the test bacterium for 24-hours.

# Analysis of biofilm inhibition

1. Following incubation and biofilm formation, use a pipette to carefully to remove the cultures from the wells, (see *Tip (c)*).
2. Gently rinse each well with sterile 10mM phosphate buffer solution (Sigma Aldrich, product no. P5244-100ML) three times to remove planktonic cells.
3. Using a multichannel pipette (Eppendorf, 12-channel variable pipette, cat no. 3125000028), transfer 200µL volumes of 2% sodium acetate (Sigma Aldrich, product no. S2889-250G) to each well to fix adherent cells to the plate surface.
4. Allow stand for thirty minutes before carefully removing the sodium acetate with a pipette. Allow the plates to dry at room temperature.
5. When dry, use the multichannel pipette to dispense 200µL volumes of 0.1% crystal violet (Sigma Aldrich, CAS no. 548-62-9) to each well. Incubate with crystal violet for ten minutes at room temperature.
6. With the multichannel pipette, carefully remove as much crystal violet as possible, (see *Tip (c)* for removing solutions from wells), before rinsing each well three times with 200µL volumes of deionised water. Allow dry completely at room temperature.
7. When dry, transfer 200µL of 95% ethanol (Sigma Aldrich, CAS no: 64-17-5) to each well. Incubating the plate at room temperature for five minutes to solubilise the crystal violet.
8. Pipette the ethanol in each well solution up and down four times before obtaining absorbance readings using an automated spectrophotometric plate reader, (Varioskan LUX multimode microplate reader, Thermo Fisher, cat no. VL0000D0), set to 595nm.
9. Compare the absorbance readings obtained from the biofilm formed in the control wells and biofilm formed in the peptide exposed wells to determine if there was a significant decrease in biofilm formation.

Perform in triplicate using three biological repeats, i.e. three separate overnight cultures of the test bacterium, to determine the standard deviation and confirm antimicrobial action.

***Tips:***

1. The addition of glucose to the culture medium has previously been found to enhance the formation of biofilm (Christensen et al., 1985; Mathur et al., 2006; Smith et al., 2016; Twomey et al., 2020). Supplement the culture medium used in the initial overnight culture and the broth used to perform the 1:100 dilution of the overnight culture with 1% glucose.
2. Resuspend the peptide at a range of concentrations to assess if the antibiofilm effects of the bacteriocin above and below the MIC. This will allow for a better assessment of bacteriocin effects on biofilm inhibition.
3. When removing any liquids from the wells, (test bacterium culture, volumes of crystal violet, sodium acetate and PBS), do so gently as not to remove bound material. If using a pipette tip, ensure that it does not scratch the sides or bottom of the wells, as this could detach biofilm, leading to an incorrect assessment of biofilm inhibition.


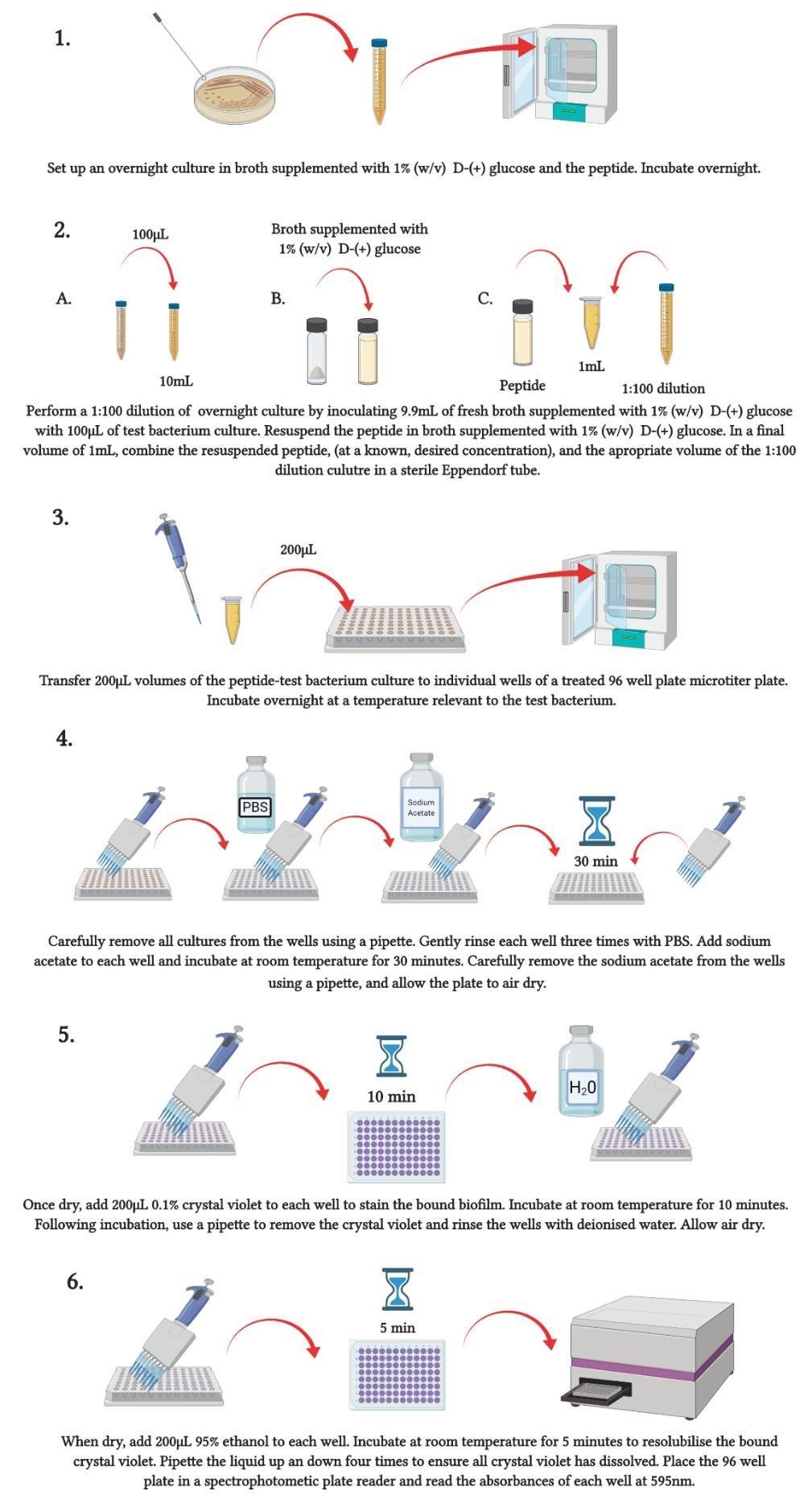


**Figure S10: Analysis of biofilm inhibition on plastic surfaces using the stationary microtiter plate method. Supplementing the culture medium with 1% (w/v) D-(+) glucose has previously been found to found to enhance biofilm production. Created using Biorender.com.**

**Protocol S13: Removal of pre-formed biofilm on plastic microtiter plates.**

# Preparing the test bacterium culture

1. Prepare a culture of the test bacterium as per **Protocol S1**, supplementing the medium with 1% (w/v) D-(+) glucose, (see *Tip (a)*), in a final volume of 10mL.

# Biofilm formation

1. Perform a 1:100 dilution on the test bacterium overnight culture by inoculating 10µL of culture into 990µL of relevant broth, supplemented with 1% (w/v) D- (+) glucose.
2. Transfer 200µL volumes of the test bacterium subculture to individual wells of a sterile 96 well microtiter plate (Round well, flat bottomed microtiter plate, with a lid; Sarstedt, order no. 82.1582.001). Ensure that a suitable number of wells are filled to facilitate both peptide exposure and controls.
3. Incubate the plate at the relevant temperature for the test bacterium strain for 24-hours.

# Removal of biofilm

1. Following incubation and biofilm formation, use a pipette to carefully to remove the test bacterium cultures from the wells, (see *Tip (b)*).
2. Using a multichannel pipette (Eppendorf, 12-channel variable pipette, cat no. 3125000028), carefully and aseptically, rinse all wells of the microtiter plate with sterile 10mM phosphate buffer solution (Sigma Aldrich, product no. P5244-100ML).
3. Resuspend the peptides in the relevant broth at the desired concentration (see *Tip (c)* for peptide concentrations).
4. Transfer 200µL volumes of the resuspended peptide to selected wells containing preformed biofilm.
5. To control wells, add 200µL of fresh, sterile broth not containing dissolved peptide.
6. Incubate the plate for another 24-hours at the relevant temperature for the test bacterium.

# Analysis of biofilm removal

1. For the analysis of biofilm formation and removal, see **Protocol S11**, steps (7) – (14).
2. Compare the absorbance readings for the control wells and the peptide exposed wells to determine if there was a significant loss of biofilm.

Perform in triplicate using three biological repeats, i.e. three separate overnight cultures of the test bacterium, to determine the standard deviation and confirm antimicrobial action.

***Tips:***

1. The addition of glucose to the culture medium has previously been found to enhance the production of biofilm (Christensen et al., 1985; Mathur et al., 2006; Smith et al., 2016; Twomey et al., 2020). Supplement the culture medium used in the initial overnight culture and the broth used to perform a 1:100 dilution of the overnight culture with 1% glucose.
2. When removing any liquids from the wells, (test bacterium cultures, volumes of crystal violet, sodium acetate and PBS), do so gently as not to remove bound material. If using a pipette tip, ensure that it does not scratch the sides or bottom of the wells, as this could detach biofilm also, leading to an improper assessment of biofilm inhibition.
3. Resuspend the peptide at a range of concentrations to assess if the biofilm removal effects of the bacteriocin above and below the MIC. This will lead to a more accurate assessment of the effects of the bacteriocin against preformed biofilm.

**Supplementary Material References:**

1. Spanu C, Scarano C, Ibba M, Pala C, Spanu V, De Santis EPL. Microbiological challenge testing for Listeria monocytogenes in ready-to-eat food: A practical approach. Ital J Food Saf. 2014;3:231–7. doi:10.4081/ijfs.2014.4518.
2. Vijayakumar P, Muriana P. Inhibition of Listeria monocytogenes on Ready-to-Eat Meats Using Bacteriocin Mixtures Based on Mode-of-Action. Foods. 2017;6:22. doi:10.3390/foods6030022.
3. Kara R, Yaman H, Gök V, Akkaya L. The effect of nisin on Listeria monocytogenes in chicken burgers. Indian J Anim Res. 2014;48:171–6. DOI: 10.5958/j.0976-0555.48.2.036
4. Hanson H, Whitfield Y, Lee C, Badiani T, Minielly C, Fenik J, et al. Listeria monocytogenes associated with pasteurized chocolate milk, Ontario, Canada. Emerg Infect Dis. 2019;25:581–4. doi:10.3201/eid2503.180742.
5. Cody SH, Glynn MK, Farrar JA, Cairns KL, Griffin PM, Kobayashi J, et al. An outbreak of Escherichia coli O157:H7 infection from unpasteurized commercial apple juice. Ann Intern Med.

1999;130:202–9. DOI: 10.7326/0003-4819-130-3-199902020-00005

1. FAO, WHO. Enterobacter sakazakii and other microorganisms in powdered infant formula.

Microbiol Risk Assess Ser. 2006;6:80. http://www.fao.org/3/a-y5502e.pdf. Accessed 4 Jul 2020.

1. Mathur T, Singhal S, Khan S, Upadhyay DJ, Fatma T, Rattan A. Detection of biofilm formation among the clinical isolates of staphylococci: an evaluation of three different screening methods. Indian J Med Microbiol. 2006;24:25–34. doi:10.4103/0255-0857.19890.
2. Twomey E, Hill C, Field D, Begley M. Bioengineered Nisin Derivative M17Q Has Enhanced Activity against Staphylococcus epidermidis. Antibiotics. 2020;9:305.

doi:10.3390/antibiotics9060305.

1. Christensen GD, Simpson WA, Younger JJ, Baddour LM, Barrett FF, Melton DM, et al. Adherence of coagulase-negative staphylococci to plastic tissue culture plates: A quantitative model for the adherence of staphylococci to medical devices. J Clin Microbiol. 1985;22:996–1006.

doi:

10.1128/JCM.22.6.996-1006.

1. Smith MK, Draper LA, Hazelhoff P-J, Cotter PD, Ross RP, Hill C. A bioengineered nisin derivative, M21A, in combination with food grade additives eradicates biofilms of Listeria monocytogenes. Front Microbiol. 2016;7:1939. doi:10.3389/fmicb.2016.01939.
